# A pair of DNA glucosyltransferases elevate counter-defense in bacteriophage T4

**DOI:** 10.1093/nar/gkag531

**Published:** 2026-05-28

**Authors:** Luis Ramirez-Chamorro, Frédéric Bonhomme, Anton Lukas Ipsen Wolff, Mathieu Stouf, François Lecointe, Marcel Hollenstein, Mart Krupovic, Marianne De Paepe, Yuvaraj Bhoobalan-Chitty

**Affiliations:** Université Paris-Saclay, INRAE, AgroParisTech, Micalis Institute, Jouy-en-Josas 78350, France; Institut Pasteur, Université Paris Cité, CNRS UMR3523, Department of Structural Biology and Chemistry, Unité de Chimie Biologique Epigénétique, 28, rue du Docteur Roux, 75724 Paris Cedex 15, France; Department of Biology, University of Copenhagen, Copenhagen N 2200, Denmark; Université Paris-Saclay, INRAE, AgroParisTech, Micalis Institute, Jouy-en-Josas 78350, France; Université Paris-Saclay, INRAE, AgroParisTech, Micalis Institute, Jouy-en-Josas 78350, France; Institut Pasteur, Université Paris Cité, CNRS UMR3523, Department of Structural Biology and Chemistry, Laboratory for Bioorganic Chemistry of Nucleic Acids, 28, rue du Docteur Roux, 75724 Paris Cedex 15, France; Institut Pasteur, Université Paris Cité, CNRS UMR6047, Cell Biology and Virology of Archaea Unit, 75015 Paris, France; Université Paris-Saclay, INRAE, AgroParisTech, Micalis Institute, Jouy-en-Josas 78350, France; Université Paris-Saclay, INRAE, AgroParisTech, Micalis Institute, Jouy-en-Josas 78350, France; Department of Biology, University of Copenhagen, Copenhagen N 2200, Denmark

## Abstract

Bacteriophages encode diverse nucleotide-modification pathways to evade host restriction-modification (RM) and CRISPR–Cas systems. On the other hand, modifications can also serve as a target for host defense systems, illustrating the complexity of the defense and counter defense landscape. Bacteriophage T4 encodes two glucosyltransferases (GTs), α-GT and β-GT, that post-replicatively add a glucose moiety to the hydroxymethylated deoxycytosines (5-hmC) on phage DNA in the α- and β-conformation, respectively. Among all fully sequenced phages, only six closely related phages encode both α-GT and β-GT. Here, through biochemical and genetic analysis, we show that β-GT has higher catalytic activity, whereas α-GT is more strongly expressed. During T4 infection, these factors determine the contributions of both GTs, with 66% of all 5-hmC α-glucosylated and 33% β-glucosylated. Encoding a single GT is sufficient to overcome the *Escherichia coli* RM systems, unless the glucosylation levels decrease below 80%, which constitute a complete protection threshold. However, when encountering a host encoding DNA glycosylase Brig1, in addition to type I and type IV RM systems, a second GT is necessary to enable Brig1 escapers to resist RM systems. These results demonstrate that encoding multiple GTs serves as a counter-defense mechanism when simultaneously confronted with several antiphage defense systems.

## Graphical Abstract

**Graphical Abstract:**
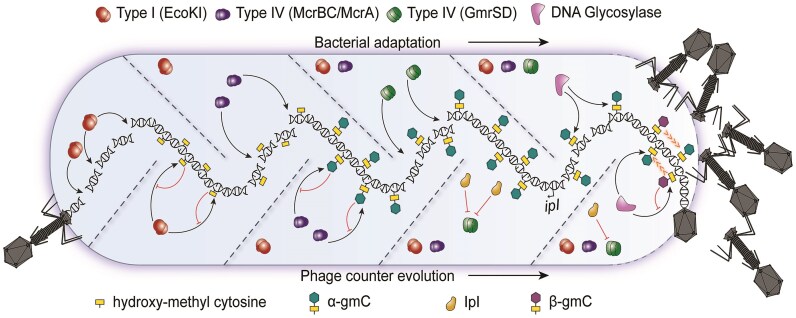

## Introduction

Bacteriophages, particularly members of the class Caudoviricetes, encode a variety of pre-replicative and post-replicative pathways to diversify their DNA nucleobase content beyond canonical composition [1]. Modifications range from methylation, the most prevalent modification among all domains of life, to complex modifications that result in hypermodified bases such as glucosylmethylcytosine (5-gmC), aminocarboxymethyladenine, and queuosine-like 7-deazaguanine derivatives [2, 3]. Methylation is a key component of the host-encoded RM systems that target viruses [4]. Similarly, hypermodification has been shown to be essential in phage T4 to evade host RM systems, as well as type I and type II clustered regularly interspaced short palindromic repeat (CRISPR)–CRISPR-associated proteins (Cas) systems [5–10]. Additionally, modifications enable recognition of non-phage (host) DNA by phage proteins, thereby contributing to transcriptional repression [11] and cleavage of host DNA by phage endonucleases [12, 13].

Hypermodified DNA bases, in particular 5-gmC, were first identified among T-even phages [14]. Phage-encoded enzymes first modify the host nucleotide precursor pool, converting deoxycytidine diphosphate and deoxycytidine monophosphate (dCMP) into hydroxymethylated deoxycytidine triphosphate nucleotides (5-hmdC) [15]. During phage replication, the modified nucleotides are incorporated into the nascent DNA. Subsequently, the glucosyl group from uridine diphosphate glucose is transferred onto the hydroxymethylated deoxycytosine (5-hmC) by glucosyltransferase (GT) enzymes [16]. In phage T4, all cytosines in DNA are exclusively mono-glucosylated by either alpha-glucosyltransferase (α-GT) or beta-glucosyltransferase (β-GT) [17, 18]. Among these, 60% possess α-glycosidic linkages (catalyzed by α-GT), while 40% have β-glycosidic linkages (catalyzed by β-GT) [19]. In T4, it has been suggested that glucosylation by α-GT is concomitant with replication and occurs upon association of the enzyme with the T4 replisome, whereas the remaining non-glucosylated 5-hmC residues serve as substrates for β-GT [20]. In phages T2 and T6, which possess only α-GT, β-glucosyl-HMC-α-GT [PF20691, hereafter referred to as TET-associated glycosyltransferase (TAGT)] catalyzes the addition of a glucosyl group onto an already α-glucosylated cytosine, resulting in 5-gentiobiosyl-methylcytosine base [17, 18, 21].

The diversification of modifications likely results from an evolutionary arms race between phages and their hosts. In the *Escherichia coli*–T4 host–phage system, hydroxymethylation of cytosine protects the phage DNA from the widely conserved type I EcoKI RM system [22]. Subsequent glucosylation protects from the methylation-specific type IV McrBC/McrA RM systems [5, 23]. DNA glycosylases such as Brig1 and the prophage-encoded type IV RM system GmrSD (glucose-modified restriction) are both capable of antiphage activity by targeting 5-gmC [24–26]. Brig1 acts specifically against α-glucosylated cytosine, whereas GmrSD targets both stereoisomers of 5-gmC and the 5-gentiobiosyl-methylcytosine. The activity of GmrSD and its variants is inhibited by phage T4 proteins IPI, IPII, and IPIII [24, 25, 27]. As an alternative to glucosylation, arabinosylation of the hydroxy deoxycytosine in the phage DNA [28, 29] also offers robust protection not only against type II RM and the DNA glycosylase Brig1 but also against type I CRISPR–Cas [30]. In addition to Brig1, antiviral glycosylases such as Dag1/Dag2 and Brig2, which cleave modified guanine and thymine bases, respectively, have also been characterized [31].

Our study reveals that while most phages encode either α-GT or β-GT, a few closely related phages, including phage T4, encode both GTs. The two phage GTs appear to have diverged from a common cellular ancestor with the GT-B structural fold following a gene duplication event, resulting in paralogous enzymes that yield stereochemically different reaction products. We show that the balanced levels of α- and β-glucosylation observed in wild-type phage is due in part to the differences in the enzymatic activity and expression strength of α-GT or β-GT. We demonstrate that each GT, when expressed alone, is sufficient to ensure >80% cytosine glucosylation, a level sufficient to provide complete protection against *E. coli* type IV restriction-modification systems. However, despite the apparent functional redundancy of the two GTs in protection against typical RM systems, we show that in the presence of the additional glycosylase-based defense system Brig1, the GT responsible for the non-targeted modifications, β-GT, serves as an efficient back-up allowing the survival of phage escaper mutants with non-targeted modifications.

## Materials and methods

### Growth conditions, PCR, plasmids, and strains

*Escherichia coli* MG1655 was utilized for the growth curve assays, involving the estimation of virulence index, and *E. coli* DH10B was utilized in genome editing experiments and plaque assays. Efficiency of plating (EOP) assays were also performed with *E. coli* MG1655, *E. coli* DH10B, *E. coli* K-12 BW25113 Δ*mcrC*, and Δ*mcrA* strains [32]. All *E. coli* cultures were grown in Luria–Bertani (LB) medium, incubated at 37°C with shaking at 200 rpm.

Oligonucleotides and PCR (polymerase chain reaction) fragments were fused together either by Overlap Extension-PCR or by Gibson Assembly (NEB #E2611S) [33] and cloned into the plasmid pBAD24. In case of the positional swapping between *α-gt* and *β-gt*, the coding sequence of β-GT was first replaced with a pseudo-gene whose sequence includes appropriate protospacers for targeting by the CRISPR–Cas13b system. A pBAD24-based plasmid was constructed with the coding sequence of α-GT fused to the homologous arms that correspond to the sequences flanking the coding sequence of β-GT. Similarly, a plasmid containing the coding sequence of β-GT flanked by the upstream and downstream sequence of α-GT was also constructed. 

5-hmC-containing DNA fragment was prepared by PCR amplification of DNA fragment using the primers T4 DNApol For and T4 DNApol Rev with T4 DNA as template. In the PCR preparation, the dNTP mixture was substituted with an alternative mixture that was prepared by mixing the individual nucleotides, dATP, dGTP, and dTTP, along with 5-hydroxymethyl-dCTP (Jena Bioscience, #NU-932S).

Spacers corresponding to target protospacers in *α-gt, β-gt*, and pseudo-gene were introduced into the plasmid pBZCas13b (Addgene #103986), encoding Cas13b, as described earlier. Briefly, overlapping oligonucleotides with 5′ extensions were pooled together at 1 μM final concentration, along with PCR reaction buffer. The mixture was incubated at 95°C for 10 min and gradually cooled to room temperature to facilitate annealing. The annealed oligonucleotides were ligated into the pBZCas13b vector linearized with the BsaI-HF (NEB #R3733S).

For heterologous expression of individual proteins, the coding sequences were cloned into the vector pET-28a(+) while retaining an in-frame C-terminal histidine sequence (in the case of *α-gt* and *β-gt*) or without an in-frame histidine coding sequence (T4 *gp45*).

All primers, plasmids, and strains used in this study are listed in Supplementary Tables S5 and S6.

### Bioinformatic analysis

Homologs of the three GTs, NP_049673.1 (α-GT, PF11440), NP_049658.1 (β-GT, PF09198), and YP_010067197.1 (β-glucosyl-HMC-α-GT/TAGT, PF20691), encoded among viruses (taxid = 10239), in the RefSeq database were identified using Position Specific Iterative BLAST (PSI-BLAST) [34], with an expect threshold = 0.005, over three iterations. The protein ids of the homologs of all three GTs were combined and organized according to their respective phage accession numbers, which was then utilized to visualize their distribution. A similar analysis was carried out against all viruses in the non-redundant (nr) NCBI GenBank database.

VICTOR (Virus Classification and Tree Building Online Resource) [35] was used to construct the phage phylogenetic tree based on phages retrieved from the RefSeq database that encode at least one GT and belong to the genus *Tequatrovirus*. Representative phages from diverse genera that also encode at least one GT were also included. Pairwise comparisons of the phage nucleotide sequences were conducted using the Genome-BLAST Distance Phylogeny method (including 100 pseudo-bootstrap replicates each) with the D0 formula for nucleotides [36]. The tree was visualized with ggtree [37]. To construct a phylogenetic tree of T6 TAGT, protein homologs were retrieved from RefSeq database during the earlier PSI-BLAST and submitted to the phylogenetic analysis tool NGphylogeny.fr with default settings [38]. The Newick tree generated by PhyML was visualized on iTOL [39].

For comparison of the six phage genomes, the complete nucleotide genome sequences of *Escherichia* phage 236Ecol005PP (PP434423.1), *Escherichia* phage FL12 (PP400786.1), *Escherichia* phage Pu-Krd-SF1 (PQ249395.1), *Escherichia* phage T4 (NC_000866.4), *Enterobacteria* phage RB55 (KM607002.1), and *Escherichia* phage vB-Eco-KMB26 (OR539218.1) were downloaded in fasta format and combined into a single file. The nucleotide sequences were then analyzed using VIRIDIC WEB [40], to calculate the nucleotide similarity between each of the six genomes, with default BLASTN parameters (‘-word_size 7 -reward 2 -penalty -3 -gapopen 5 -gapextend2’).

### Three-dimensional structure prediction and analysis

PDB identifiers for representatives of different GT families were retrieved from the CAZy database [41]. All viral and cellular protein structures were downloaded from the PDB database [42]. Structural models of phage proteins for which experimentally determined structures were not available were either downloaded from the BFVD database [43] or modeled using AlphaFold3 [44]. Structural similarities between cellular and viral proteins were evaluated based on the DALI Z score, which is a measure of the quality of the structural alignment. Z scores above 2, i.e. 2 SDs above expected, are usually considered significant [45]. Structural similarity matrix from all-against-all structure comparisons as well as corresponding dendrograms were obtained using the latest release of the DALI server [46]. Structures were visualized using the University of California, San Francisco (UCSF) ChimeraX v1.9 [47].

### Protein purification

Two-hundred milliliter cultures of *E. coli* BL21(DE3) strains (Novagen) carrying pET-28a(+)_*α-gtchis* or pET-28a(+)_*β-gtchis* were grown in LB medium supplemented with kanamycin (50 μg/ml) at 37°C, 200 rpm until optical density (OD_600_) = 0.6–0.8 was reached. Cultures were then incubated at 30°C for 3 h and protein expression induced by the addition of isopropyl-β-D-thiogalactopyranoside (IPTG) to a final concentration of 0.5 mM. Cells were collected by centrifugation at 6300 × *g* for 10 min at 16°C. The pellets were resuspended in lysis buffer (25 mM Tris–Cl, pH 7.5, 200 mM NaCl, 5% glycerol, and 30 mM imidazole) and stored at −16°C until further processing. The thawed mixture was lysed by homogenization (STANSTED model SPCH-10 from homogenizing systems, UK) and sonication (30 cycles, 3 s on and 3 s off). The cell debris was removed by centrifugation at 12 000 × g for 30 min at 4°C and the supernatant was filtered with a 0.45-μM filter. Proteins were bound to histrap columns (HisTrap™ High Performance column, Cytiva), equilibrated with the lysis buffer. After washing with 40 column volumes of the lysis buffer including 30 mM imidazole, the proteins were eluted in a buffer including 500 mM imidazole. The eluted fractions, containing the protein of interest, were pooled together and concentrated using Pierce™ Protein Concentrators PES 10K MWCO (Thermo Fisher Scientific, #88 516). The concentrated fractions were loaded on a Superdex 200 Increase 10/300 GL column (Cytiva) equilibrated in 25 mM Tris–Cl (pH 7.5), 200 mM NaCl, and 5% glycerol (SEC buffer).

### Analysis of α-GT-gp45 and β-GT-gp45 interactions, *in vitro*

For the analysis of *in vitro* protein–protein interaction, 500 ml cultures of *E. coli* BL21(DE3) strains carrying pET-28a(+)_*gp45* or pET-28a(+)_*α-gtchis* or pET-28a(+)_*β-gtchis* were grown in LB medium supplemented with kanamycin (50 μg/ml) at 37°C, 200rpm until OD_600_ = 0.4–0.6 was reached. Cultures were then incubated overnight at 16°C and protein expression induced by the addition of IPTG to a final concentration of 0.5mM. Cell pellets were collected by centrifugation at 6300 × g for 10 min at 16°C. The pellets were resuspended in lysis buffer (25mM Tris–Cl, pH 7.5, 150 mM NaCl, and 5% glycerol). Cell lysis was achieved as described above. The filtered supernatant containing tag-free gp45 was split into two fractions and mixed with supernatants containing α-GT chis or β-GT chis. The supernatants were then combined with Ni-NTA Agarose (Qiagen, #30 210), equilibrated with the lysis buffer. The mixtures were incubated overnight at 4°C. Prior to elution, the agarose beads were washed three times with 20 column volumes of lysis buffer containing 20 mM imidazole and eluted in 1 column volume of buffer including 250 mM imidazole. Filtered supernatants of the individual proteins were also treated similarly and purified, as controls.

### Competition assay

Substrate DNA containing 5-hmC was amplified using the primers T4 DNApol For and T4 DNApol Rev as described earlier. The glucosylation reaction was carried out in NEB buffer 4 and included 740 ng of DNA (equivalent to 400 pmol of 5-hmdC, 16 pmol/μl final concentration) and Uridine-5′-diphosphoglucose disodium salt (MP Biomedicals, LLC, cat no. 101208) to a final concentration of 2 μM, along with the appropriate amounts of GT to a final concentration of 1.5 pmol/μl. The reactions were incubated at 37°C for 60 min, following which the products were directly digested using the nucleoside digestion mix and quantified by tandem mass spectrometry coupled to liquid chromatography (LC-MS/MS) (described below).

### Phage preparation and plaque assay

Phage stocks were prepared as lysates by infection of *E. coli* DH10B cultures in exponential phase (OD_600_ = 0.1–0.2) in LB with 10 mM MgSO_4_ and 5 mM CaCl_2_. After the infected cultures reached an OD_600_ <0.1, they were centrifuged and the supernatant was filtered. The stocks were titrated and stored at 4°C until use.

For plaque assay, preheated top agar (0.4% LB-agar) was mixed with 100 μl of overnight culture and phage preparation. The mixture was then poured onto preheated LB agar plates and incubated overnight at 37°C. The plaques were counted and the plaque-forming unit per milliliter (PFU/ml) was calculated.

### Genome editing of phage T4

Insertions and deletions in bacteriophage T4 were performed as described earlier [48]. Briefly, overnight cultures of the recombinant strains, *E. coli* carrying plasmids constructed for deletions or insertions, were diluted and incubated at 37°C until an OD_600_ between 0.15 and 0.25 was reached. The cultures were then infected with phage T4 preparations at an MOI (multiplicity of infection) of 0.05 and incubated at 37°C until complete lysis was observed (1–2 h post infection). Serial dilutions of the supernatants were spotted on LB plates with the top layer containing the selection strain, *E. coli* DH10B with spacer containing pBZCas13b plasmid, to target the non-recombinant phage T4. The overnight-incubated plates were then screened for phage mutants with the appropriate mutation by PCR.

Although replacement of the α-GT coding sequence with *β-gt*, resulting in a recombinant phage with two copies of *β-gt*, is possible to construct using CRISPR–Cas-based genome editing, subsequent replacement of the native β-GT coding sequence with *α-gt* becomes unachievable, as the CRISPR spacer would target both *β-gt* copies. Instead, we developed an alternative strategy in which we replaced the β-GT coding sequence with a pseudo-open reading frame (pseudo-ORF) in wild-type T4 phage, to construct phage T4 LySE. The pseudo-ORF carried three protospacers, designed for efficient targeting by the CRISPR–Cas13b system (Supplementary Fig. S8A). Utilizing spacers designed for targeting *α-gt* transcripts, we introduced the β-GT coding sequence downstream of the *α-gt* promoter (referred to as T4∇P*_α-gt_::β-gt*). Subsequently, this recombinant phage was further engineered to replace the pseudo-ORF with the *α-gt* coding sequence downstream of the *dCMP*/*β-gt* promoter (referred to as T4∇P*_α-gt_::β-gt* P*_β-gt_:: α-gt*) (Fig. 4B and Supplementary Fig. S8B). Another phage with single replacements was also constructed, referred to as T4∇P*_β-gt_::α-gt*, in addition to T4∇P*_α-gt_::β-gt*.

### Quantification of nucleosides by LC-MS/MS

Total phage genomic DNA was extracted as described earlier [48]. Phage suspension was mixed with PEG and NaCl to a final concentration of 10% and 0.5 M, respectively, and incubated overnight at 4°C. Phages were pelleted by centrifugation at 12 000 rpm for 30 min at 4°C and resuspended in SM buffer (50 mM Tris–HCl, pH 7.5, 100 mM NaCl, 8 mM MgSO_4_). Total DNA was extracted using Phenol–Chloroform followed by ethanol precipitation. One microgram of total DNA was digested in 20-μl reaction volume using the NEB Nucleoside digestion mix (#M0649S) according to the manufacturer’s instructions.

Analysis of global levels of dC, 5-mdC, 5-hmdC, 5(α)-gmdC, and 5(β)-gmdC were performed on a Q Exactive Orbitrap Mass Spectrometer (Thermo Fisher Scientific) equipped with an electrospray ionization source (H-ESI II Probe) coupled with an Ultimate 3000 RS HPLC (Thermo Fisher Scientific). Digested DNA was injected into Thermo Fisher Hypersil GOLD aQ chromatography column (100 mm × 2.1 mm, 1.9 μm particle size) heated at 30°C. The flow rate was set at 0.3 ml/min and run with an isocratic eluent of 1% ACN in water with 0.1% formic acid for 10 min. Parent ions were fragmented in positive ion mode with 10% normalized collision energy in parallel-reaction monitoring mode. MS2 resolution was 17 500 with an AGC target of 2e5, a maximum injection time of 50 ms, and an isolation window of 1.0 *m*/*z*. The inclusion list contained the following masses: dC (228.1), 5-mdC (242.1), 5-hmdC (258.1), and 5(α)-gmdC/5(β)-gmdC (420.2). Extracted ion chromatograms of base fragments (±5 ppm) were used for detection and quantification (112.0506 Da for dC; 126.0662 Da for 5-mdC; 142.0609 Da for 5-hmdC; and 304.1133 Da for 5(α)-gmdC/5(β)-gmdC). 5(α)-gmdC and 5(β)-gmdC were distinguished by their retention times. Calibration curves were previously generated using synthetic standards (Clinisciences, France) in the ranges and 0.01–1 pmol.

### Fluorescence microscopy

*Escherichia coli* MG1655 cultures, prepared from a 200-fold dilution of overnight culture, were grown to an OD_600_ of 0.6 in LB at 37°C and infected with T4 mutant phage T4Δ*α-gt*∇P*_α-gt_::mVenusNB* or T4Δ*β-gt*∇P*_β-gt_::mVenusNB*. Immediately following infection, the cell and phage mix was spread on a 1% agarose LB pad prepared on a microscope slide, using a Thermo Scientific™ Gene Frame (#11550294). Images were acquired at 37°C using an inverted Nikon Eclipse Ti-E micrscope equipped with a KINETI sCMOS camera, a CoolLED pE-800 light source with LED wavelength at 500 nm for YFP, a Plan APO X100 oil immersion objective (N.A. 1.45), and an Okolab temperature-controlled chamber. YFP exposure time and intensity were 500 ms and 5%, respectively. Images were taken every 2 min for 1 h and frames at 4, 14, and 24 min were selected for analysis. Image analysis was performed with OUFTI^©^ software [49], using the cytoplasmic YFP signal for cell segmentation. Mean fluorescence intensities from the YFP channel, for lysing cells, were extracted and plotted using Matlab Mathworks^©^ suite.

### Virulence index

Virulence index was calculated as previously reported [50]. Briefly, 300 µl overnight bacterial culture was used to inoculate 30 ml of LB, which was then incubated for 1 h at 37°C with agitation at 200 rpm. Once the cultures reached OD_600_ between 0.15 and 0.25, 100 µl was distributed in a 96-well plate. In parallel, pre-titrated phage lysates were serially diluted and 100 µl of each dilution was added to each well, covering a range of MOIs from 10^2^ to 10^−6^. The OD_600_ of the cultures was measured every 5 min over 16 h to obtain bacterial reduction curves.

The limit of integration was set at 7 h post infection, since beyond this time point growth of phage-resistant bacteria was observed. Area under the curve (AUC) of the bacterial reduction ones were obtained through the function MESS::auc depicted as local virulence curves [AUC versus log_10_(MOI)]. The virulence index of each phage was obtained by integrating the virulence curves and normalizing. Virulence index values were compared by Kruskal Wallis Pairwise comparison and Dunn’s all-pairs test.

### Efficiency of plating assay

EOP was estimated through plaque assays. Ten-fold serial dilutions were prepared from a single stock of the phages. Ten microliters of phage dilution and 100 μl of overnight bacterial culture was mixed with preheated top agar (0.4% LB-agar) and poured on LB agar plates. After overnight incubation at 37°C, the number of plaques was counted and the PFU/ml was determined for both the test and control strains. EOP was calculated as the ratio of their PFU/ml values.

## Results

### Co-occurrence of α- and β-glucosyltransferases is rare in phage genomes

**Figure 1.**
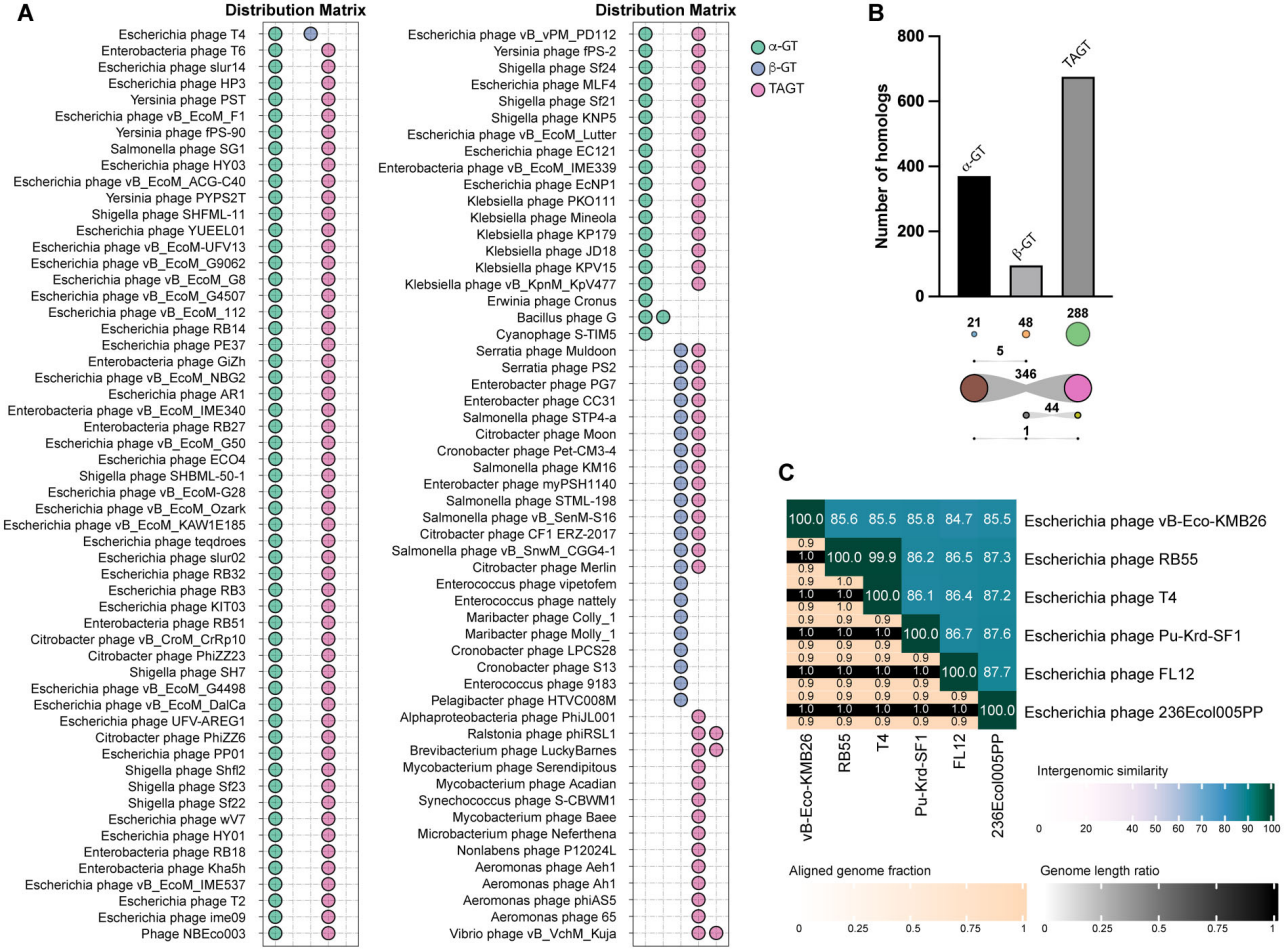
Distribution of GTs among phage genomes. (**A**) Distribution of α-GT, β-GT, and TAGT homologs in phage genomes from the RefSeq database. (**B**) Bar-plot showing the total number of α-GT, β-GT, and TAGT homologs in viruses (taxid:10239) in the nr NCBI GenBank database. The number of GT homologs encoded either individually or in combination with other GT(s) is indicated below the *x*-axis. Circle sizes are proportional to the number of phages encoding either individual GTs or their combinations (when linked by a gray area). (**C**) Heatmap showing pairwise sequence similarities between the six Escherichia phages that encode both α-GT and β-GT. The top-right half of the heatmap, above the diagonal, displays the percentage of similarity between each genome pair. In the bottom-left half, three values are provided for each pair: the top and bottom values represent the aligned fraction for the phage genome pair corresponding to that row and column, respectively. The middle value is the ratio of their genome lengths.

Genetic redundancy is rare in phage genomes, due to a high constraint on maximal genome content [51–53]. Yet β-GT in phage T4 was previously described to have a redundant function with α-GT, which is intriguing [54]. To investigate this phenomenon, we started by analyzing the distribution of GTs in phage genomes. We first identified all viral homologs of T-even phage GTs in the RefSeq database by PSI-BLAST (Supplementary Table S1). The hits were then associated to their respective phage genomes (Fig. 1A and Supplementary Table S2). Among the 5388 phage genomes in the RefSeq database, 111 phages encode GT homologs of which 56 phages carried two types of GT. In all cases, except phage T4 and *Bacillus* phage G, phage genomes contained α-GT or β-GT alone, or in association with a homolog of a TAGT. Bacillus phage G contains a second copy of α-GT, whereas phage T4 is unique in encoding both α-GT and β-GT.

To check to what extent the co-occurrence of α-GT and β-GT in T4 is an exception, we expanded our analysis to the nr database, which comprises all sequenced phage genomes of the GenBank database. Among the 753 phages that carried at least one homolog of T-even phage GTs, only 1 encoded all three GT types, and an additional 5 phages, including phage T4, encoded both α-GT and β-GT (Fig. 1B and Supplementary Table S3). The overall nucleotide identity between the six phage genomes ranged from 84% to 88%, with a coverage of 90%–100% (Fig. 1C), indicating that these phages are closely related and belong to the same *Tequatrovirus* genus. A whole-genome tree showed that the five phages encoding both α-GT and β-GT share a common ancestor (Supplementary Fig. S1). This analysis also confirmed that the vast majority of phage genomes carry either α-GT or β-GT in association with a TAGT. Surprisingly, several phages also encode distantly related TAGTs in the absence of any recognizable homologs of α-GT or β-GT (Supplementary Fig. S2). Collectively, these findings indicate that the co-occurrence of α-GT and β-GT is an uncommon event, which may indicate that either it does not confer a selective advantage to the phage or the acquisition of a second GT, resulting in their co-occurrence, is a relatively recent event in phage evolution.

### Evolutionary relationship between α-GT, β-GT, and TAGT

**Figure 2.**
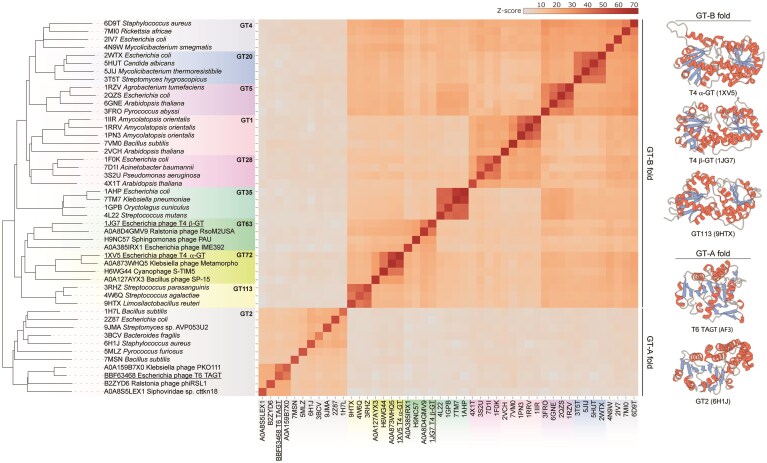
Relationships between α-GT, β-GT, and TAGT. A dendrogram and heatmap based on pairwise Z-scores, obtained using DALI, comparing T4 α-GT, T4 β-GT, and modeled structure of TAGT along with representative cellular GTs from diverse families of the GT-A and GT-B superfamilies. Experimentally determined structures and structural models are indicated with the corresponding PDB and UniProt accession numbers, respectively. α-GT and β-GT of phage T4 and TAGT of phage T6 are underlined. Different GT families are highlighted with different background colors on the dendrogram. The color scale indicates the corresponding Z scores. On the right, the crystal structures of T4 α-GT (PDB: 1XV5), T4 β-GT (PDB: 1JG7), and the modeled structure of TAGT (AF3) are compared to the structures of closest cellular representatives from the GT-B and GT-A fold superfamilies GT113 (PDB: 9HTX) and GT2 (PDB: 6HI1J), respectively. Structural models are colored according to the secondary structure elements: α-helices, red; β-strands, blue; random coil, gray.

To investigate this hypothesis, we determined the evolutionary relationships between the three GT types. Homologs of the α-GT, β-GT, and TAGT do not display any appreciable sequence similarity across the GT types (Supplementary Table S1). However, structural comparison showed that α-GT and β-GT adopt the same structural fold [55, 56], known as the GT-B [57] (Fig. 2), suggesting that the two enzymes are evolutionarily related. By contrast, analysis of the predicted structural model of TAGT from phage T6 generated with AlphaFold3 revealed that it belongs to an evolutionarily distinct superfamily of GTs that adopt the GT-A fold [57] (Fig. 2). To gain further insights into their origins and evolution, we performed structural clustering of the phage GTs with representatives from diverse GT families using DALI [46]. α-GTs and β-GTs represent two distinct GT families GT63 and GT72, respectively, which are exclusively found in phages [41]. However, our analysis showed that in structural comparisons, the two families clustered together, forming sister groups in the structure-based dendrogram (Fig. 2). Notably, among other GT-B superfamily members, the phage GTs were most closely related to the bacterial GT113-family GTs (Fig. 2). These results suggest that α-GT and β-GT have evolved following gene duplication from a common ancestor, which itself has originated from a cellular GT. TAGT homologs from phages clustered together as a sister group to the GT2 family GTs of the GT-A fold superfamily, also suggesting cellular ancestry of the phage TAGTs (Fig. 2).

Together, these results suggest that cellular GTs have been co-opted for hypermodification of the phage DNA at least twice independently (α-GT/β-GT and TAGTs, respectively). In the case of α-GT and β-GT, it is tempting to speculate that specificity toward the DNA substrate has evolved once, in the common ancestor of the two families, followed by gene duplication and diversification of the paralogs toward different stereospecificities.

### Protein–protein interaction, relative enzymatic activity, and expression strength govern the distribution of glycosidic linkages

**Figure 3.**
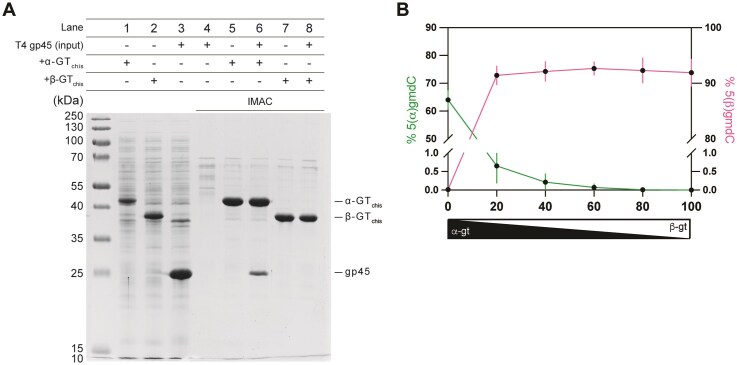
Protein–protein interaction and enzymatic analysis of α-GT and β-GT. (**A**) *In vitro* pull-down of histidine tagged α-GT or β-GT mixed with un-tagged T4gp45. Lane 1, lane 2, and lane 3 correspond to the total cell extract of *E. coli* BL21(DE3) carrying plasmid-borne *α-gtchis, β-gtchis*, and un-tagged *T4gp45* after induction with IPTG. Lane 4 is a control IMAC purification of un-tagged gp45. Lanes 5 and 6 are IMAC purification of C-terminal histidine tagged α-GT without or with fraction containing tag-free T4gp45 (shown in lane 3). Lanes 7 and 8 are IMAC purification of C-terminal histidine tagged β-GT without or with fraction containing tag-free T4gp45 (show in lane 3). (**B**) Competition assay to estimate the relative rate of glucosylation by α-GT and β-GT on a fragment of DNA containing 5-hmC. The average percentage of 5(α)-gmdC and 5(β)-gmdC nucleotides were estimated by mass-spectrometry. The values shown are average of six independent experiments, represented as mean ± SD.

Before addressing the potential advantage of encoding two GTs with different stereospecificities, we investigated the mechanisms behind the distribution of α- and β-glycosidic modifications on cytosines in T4 DNA. Previously it has been estimated that α-GT and β-GT contribute, respectively, to 60% and 40% of glucosylation [17, 19]. To explain that distribution, it has been previously hypothesized, based on *in vitro* results [20], that T4 α-GT binds to gp45, the sliding clamp protein that forms the basis of the T4 replication machinery, and modifies the T4 DNA concomitantly with replication, with β-GT presumed to intervene latter, on unmodified bases. To investigate this possibility, we analyzed the interactions between the sliding clamp protein and α-GT or β-GT. Tag-free gp45 was overexpressed in *E. coli* (Supplementary Fig. S3). Soluble fractions containing either C-terminally histidine tagged α-GT or β-GT were combined with the fraction containing gp45 prior to immobilized metal affinity chromatography (IMAC) purification. Compared to the control from which the gp45-containing fraction was omitted, a protein band matching the size of gp45 was observed in the eluted fractions of α-GT (Fig. 3A), substantiating the published results [20]. Yet, a similar observation was not made with the eluted fractions of β-GT.

Although this result supports the hypothesis that the dominance of α-glucosylation is due to the affinity of α-GT for gp45, we investigated other possible contributing factors of glucosylation levels, including potential differences in the expression of the two GTs and/or their enzymatic activity.

We first estimated the difference in enzymatic activity between the two GTs with a competition assay. Based on the elution volume during size-exclusion chromatography of histidine tagged α-GT and β-GT, it was estimated that both proteins exist as monomers (Supplementary Fig. S4). The two GTs were mixed at different molar ratios and incubated with a PCR fragment containing 5-hmC. The contribution of each GT was estimated from the percentage of 5-hmC residues that were either α- or β-glucosylated. In the absence of β-GT, α-GT glucosylated 64% of 5-hmC bases. Upon addition of β-GT, even at a concentration four times lower than that of α-GT, 91% of 5-hmC were β-glucosylated, with the α-GT contributing to only 0.65% of glucosylations (Fig. 3B). These results demonstrate that under the *in vitro* conditions used, β-GT exhibits much higher enzymatic activity than α-GT, despite contributing to only 40% of all glucosylations *in vivo*. This strongly suggests that the higher level of glucosylation by α-GT in T4 phage DNA is not due to its higher enzymatic activity.

**Figure 4.**
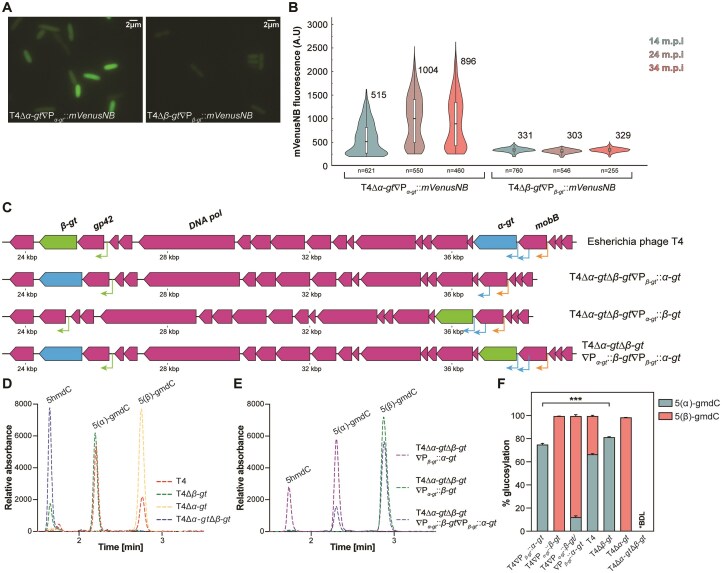
Contribution of expression strength in competitive glucosylation by α-GT and β-GT. (**A**) Fluorescence microscopy images of *E. coli* MG1655 cells infected with phage T4Δ*α-gt*∇P*_α-gt_::mVenusNB* (left panel) or T4Δ*β-gt*∇P*_β-gt_::mVenusNB* (right panel). (**B**) Violin distribution plot of the mean fluorescence intensities at 14, 24, and 34 min post infection (m.p.i) of *E. coli* MG1655 infected with phages T4Δ*α-gt*∇P*_α-gt_::mVenusNB* or T4Δ*β-gt*∇P*_β-gt_::mVenusNB*. The mean values are depicted next to the plots and the number of cells analyzed (*n*) is shown on the *y*-axis. (**C**) Illustration of phage mutants with genomic location of *α-gt* and *β-gt* swapped between their native locations in the wild-type T4 phage. (**D**) Mass-spectrometric LC-MS/MS profile of the mutant phages, T4Δ*α-gt*, T4Δ*β-gt* and T4Δ*α-gt*Δ*β-gt*, in comparison to the wild-type T4 phage. All phages were propagated in *E. coli* DH10B prior to estimation of their nucleotide composition. The curves show the abundance of 5-hmdC, 5(α)-gmdC, and 5(β)-gmdC nucleotides. (**E**) Mass-spectrometric LC-MS/MS profile of the mutant phages T4∇P*_α-gt_::β-gt*, T4∇P*_β-gt_::α-gt*, and T4∇P*_α-gt_::β-gt*/P*_β-gt_::α-gt* propagated in *E. coli* DH10B. The curves show the abundance of 5-hmdC, 5(α)-gmdC, and 5(β)-gmdC nucleotides. (**F**) Average percentage of 5(α)-gmdC and 5(β)-gmdC in phages T4∇P*_α-gt_::β-gt*, T4∇P*_β-gt_::α-gt*, T4∇P*_α-gt_::β-gt*/P*_β-gt_::α-gt*, T4, T4Δ*α-gt*, T4Δ*β-gt*, and T4Δ*α-gt*Δ*β-gt*, as estimated by mass spectrometric analysis. The values shown are from three biological replicates, represented as mean ± SD. A two-tailed unpaired *t*-test of the percentage values of 5(α)-gmdC was used to calculate the *P-*values; ***P < .001.

We then wondered whether the higher α-glucosylation levels could also stem from higher expression of *α-gt* during the T4 lytic cycle. *α-gt* is part of a polycistronic transcript that includes the upstream *mobB* gene regulated by a T4 middle promoter. In addition, two T4 middle promoter-like elements are located at the 3′ end of *mobB* and in the intergenic region between *mobB* and *α-gt. β-gt* is also part of a polycistronic transcript that includes the upstream *gp42* (dCMP hydroxymethylase), regulated by a T4 middle promoter (Supplementary Fig. S5). An analysis of previously published transcriptomics data [58] showed no significant differences in the expression levels of the two GTs, despite slight differences in the timing of expression (Supplementary Fig. S6). To compare the native expression strengths and dynamics of the two GTs with more precision than in the transcriptomic analysis, we first constructed two mutant phages, T4Δ*α-gt*∇P*_α-gt_::mVenusNB* and T4Δ*β-gt*∇P*_β-gt_::mVenusNB* (Supplementary Fig. S7), expressing the fluorescent protein mVenusNB downstream of P*_α-gt_* and P*_β-gt_*, respectively. Following infection with the mutant phages, fluorescence levels of *E. coli* MG1655 cells were tracked by microscopy (Fig. 4A). The mean fluorescence of T4Δ*β-gt*∇P*_β-gt_::mVenusNB* infected cells remained constant throughout the infection cycle whereas cells infected with T4Δ*α-gt*∇P*_α-gt_::mVenusNB* showed increasing fluorescence with progression of the phage life cycle. The mean fluorescence intensity observed upon infection with the mutant phage T4Δ*α-gt*∇P*_α-gt_::mVenusNB* was between 1.5- and 3-fold higher than that upon infection with phage T4Δ*β-gt*∇P*_β-gt_::mVenusNB* (Fig. 4B), conclusively demonstrating that the expression strength of P*_α-gt_* is greater than that of P*_β-gt_*, especially in the second part of the lytic cycle.

To determine the impact of expression strength on glucosylation level, and rule out potential post-translational effects that could differentially affect either α-GT or β-GT amounts, we then set out to modify the transcription levels of *α-gt* and *β-gt*. Due to the technical challenges associated with direct promoter modification on phage genome and lack of knowledge on the ensuing impact on expression strength, we opted instead to switch the positions of the two GTs. We constructed the mutant phage T4Δ*α-gt*Δ*β-gt*∇P*_α-gt_::β-gt*∇P*_β-gt_::α-gt* (referred to as T4∇P*_α-gt_::β-gt*∇P*_β-gt_::α-gt*), encoding both GTs but under the control of each other’s regulatory sequences, along with two additional phage mutants T4Δ*α-gt*Δ*β-gt*∇P*_β-gt_::α-gt* (referred to as T4∇P*_β-gt_::α-gt*) and T4Δ*α-gt*Δ*β-gt*∇P*_α-gt_::β-gt* (referred to as T4∇P*_α-gt_::β-gt*), encoding either α-GT or β-GT under the control of the promoter of the other GT (Fig. 4C and Supplementary Figs S8 and S9, see the “Materials and methods” section).

We then evaluated the impact of the switching of promoters on the relative levels of α- and β-glucosylated cytosine residues by LC-MS/MS. In wild-type T4, we found that 66% of all 5-hmC bases were α-glucosylated and the remaining 33% were β-glucosylated (Fig. 4D and F), in close agreement with the previous estimations [17, 19]. By contrast, in T4∇P*_α-gt_::β-gt*∇P*_β-gt_::α-gt*, while 100% of 5-hmC were glucosylated like in the T4 wild type, only 12% were α-glucosylated, with the remaining 87% being β-glucosylated (Fig. 4E and F). Thus, expression of *β-gt* from the P*_α-gt_* promoter in phage T4∇P*_α-gt_::β-gt*∇P*_β-gt_::α-gt* lowers the contribution of α-GT by seven-fold. This indicates that the high expression strength of P*_α-gt_* results in greater contribution toward glucosylation of the enzyme whose expression it controls. We also compared the glucosylation levels in phages encoding a single GT, either single-deletion mutants [48] or the phages with the switched promoters described above. In phages encoding only β-GT, either T4Δ*α-gt* or T4∇P*_α-gt_::β-gt*, 99% of 5-hmC were β-glucosylated (Fig. 4D–F). Yet, in α-GT-encoding phages, 75% of 5-hmC were α-glucosylated with P*_β-gt_::α-gt*, while 80% α-glucosylation was observed with the native P*_α-gt_::α-gt* of phage T4Δ*β-gt* (Fig. 4D–F), a small but significant difference (*P*-value = .0004), further substantiating the higher strength of P*_α-gt_* compared to P*_β-gt_*. In a double *gt* deletion mutant that was generated in this study (Supplementary Fig. S10), as expected, all cytosines were only hydroxymethylated.

Together, these results indicate that despite the higher enzymatic activity of β-GT, the stronger upstream regulatory sequences and/or the Shine-Dalgarno motif associated with *α-gt*, and not the interaction of its gene product with the sliding clamp protein of the T4 replication machinery, is mostly responsible for α-glucosylation being the predominant modification.

### Each individual GT is sufficient to provide wild-type level virulence

To investigate the potential selective advantage of encoding both α-GT and β-GT, as a first step, we characterized the virulence of T4 GT deletion mutants in relation to their percentage of cytosine glucosylation. To this end, we estimated the killing activity of the wild type, single-, and double-deletion mutant phages by determining their virulence index (V*_P_*), a recently proposed quantitative measure of the virulence of a phage against a given host [50].

**Figure 5.**
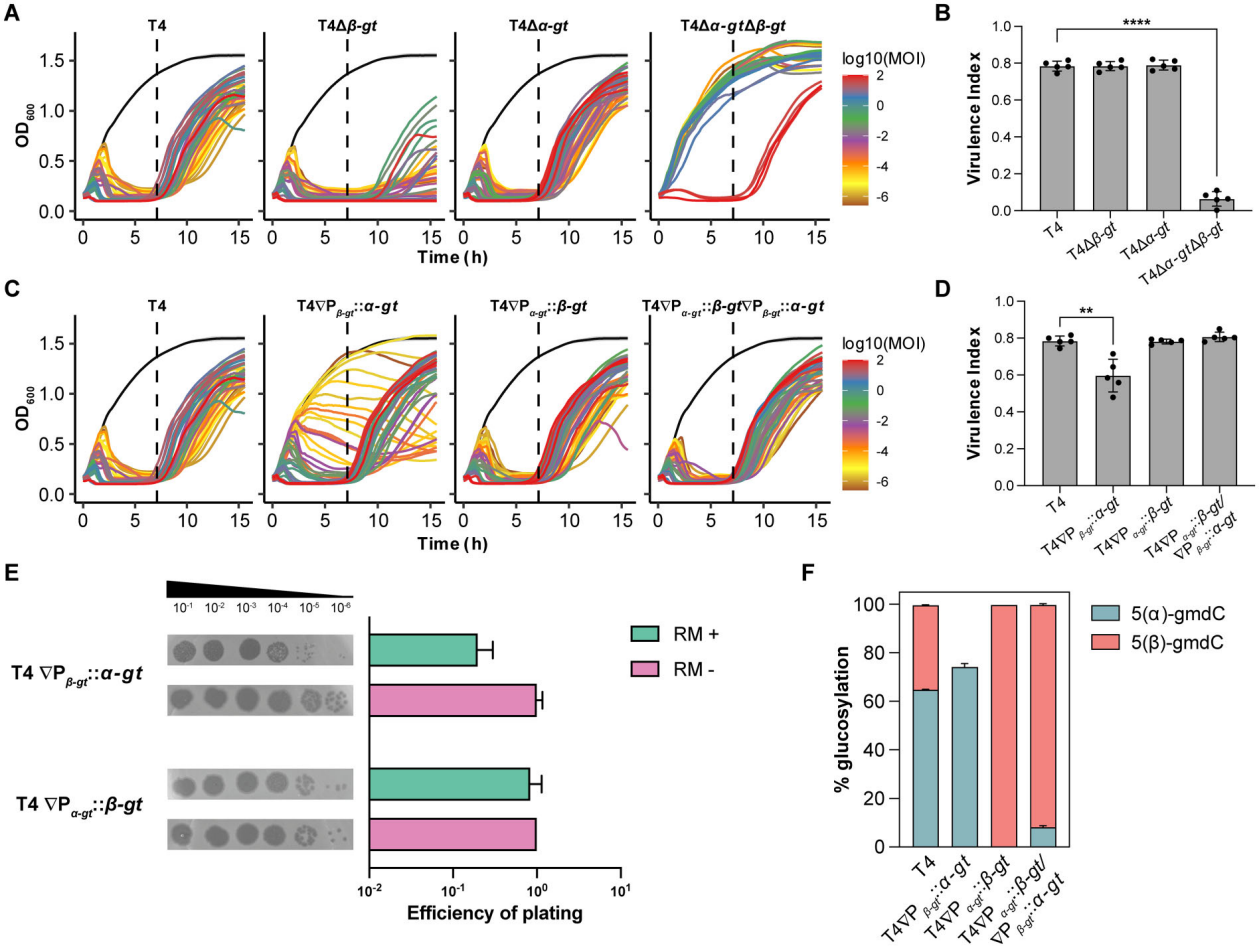
Virulence estimation of wild-type T4 phage and its mutants in *E. coli* MG1655. (**A**) Growth curves of *E. coli* MG1655 infected with phages T4, T4Δ*α-gt*, T4Δ*β-gt*, and T4Δ*α-gt*Δ*β-gt*, over a range of MOIs between 10^2^ and 10^−6^. Uninfected culture is represented in black. (**B**) Virulence index of the respective phages, as calculated from the growth curves shown in panel (A), up to 7 h post-infection. Data shown are mean of four biological replicates, represented as mean value ± SD. A two-tailed unpaired *t*-test of the virulence index values was used to calculate the *P-*values; ****P < .0001. (**C**) Growth curves of *E. coli* MG1655 infected with phages T4∇P*_α-gt_::β-gt*, T4∇P*_β-gt_::α-gt*, and T4∇P*_α-gt_::β-gt*/P*_β-gt_::α-gt*, and the wild-type T4, over a range of MOIs between 10^2^ and 10^−6^. Uninfected culture is represented in black. (**D**) Virulence index of the respective phages, as calculated from the growth curves shown in panel (C), up to 7 h post-infection. Data shown are mean of four biological replicates, represented as mean value ± SD. A two-tailed unpaired *t*-test of the virulence index values was used to calculate the *P-*values; **P < .01. (**E**) Spot assay of serially diluted phages T4∇P*_α-gt_::β-gt* and T4∇P*_β-gt_::α-gt* on *E. coli* DH10B (RM−) or *E. coli* MG1655 (RM+) background. EOP of phage infection, as estimated from the spot assay, are from four biological replicates, represented as mean ± SD. (**F**) Average percentage of 5(α)-gmdC and 5(β)-gmdC in phages T4, T4∇P*_α-gt_::β-gt*, T4∇P*_β-gt_::α-gt*, and T4∇P*_α-gt_::β-gt*∇P*_β-gt_::α-gt*, as estimated by mass spectrometric analysis. All phages were propagated in *E. coli* MG1655 prior to estimation of their nucleotide composition. The values shown are from two biological replicates, represented as mean ± SD.

Except for the double-deletion mutant, T4Δ*α-gt*Δ*β-gt*, the WT, and single-deletion mutants were able to lyse host cultures at all initial MOIs (Fig. 5A). In congruence, V*_P_* estimated over 7 h of growth post infection was similar between these phages, but the double mutant had a much lower V*_P_* (Fig. 5B and Supplementary Fig. S11). To establish the role of the individual type IV RM systems in inhibiting the double-deletion mutant, we estimated the EOP of the mutant phages in *mcrA* and *mcrC* host mutants. Aligned with the results of the virulence index assay in RM+host, only propagation of the double-deletion mutant, T4Δ*α-gt*Δ*β-gt*, was inhibited in hosts encoding either type IV RM systems, with McrBC exhibiting at least 15-fold higher inhibition compared to McrA (Supplementary Fig. S12).

To evaluate the impact of the glucosylation levels obtained with switched promoters on phage virulence, growth of the three mutant phages on *E. coli* MG1655 was compared to that of the wild-type phage (Fig. 5C). While the mutant phages T4∇P*_α-gt_::β-gt* and T4∇P*_α-gt_::β-gt*∇P*_β-gt_::α-gt* demonstrated the same levels of virulence as the wild-type phage, the mutant phage T4∇P*_β-gt_::α-gt* showed decreased virulence, evident at lower MOIs (Fig. 5D and Supplementary Fig. S13). Spot assay of the mutant phages T4∇P*_α-gt_::β-gt* and T4∇P*_β-gt_::α-gt* on *E. coli* DH10B (no RM systems) and *E. coli* MG1655 reinforced this observation: in addition to a reduced plaque size, a five-fold decrease in the EOP was observed only with phage T4∇P*_β-gt_::α-gt* in the presence of RM systems (Fig. 5E).

In light of the glucosylation levels observed in the single *gt* deletion mutants, the decreased virulence observed in phage T4∇P*_β-gt_::α-gt* suggests that 80% glucosylation, observed in T4Δ*β-gt*, is the absolute threshold below which phage DNA is susceptible to the activity of the *E. coli* type IV RM systems. Previous estimations of glucosylation level were performed using genomic DNA obtained from phages propagated in an RM-negative strain (*E. coli* DH10B) (Fig. 4E). To determine whether the presence of the RM system influences the glucosylation level, the analysis was repeated for phages T4, T4∇P*_β-gt_::α-gt*, T4∇P*_α-gt_::β-gt*, and T4∇P*_α-gt_::β-gt*∇P*_β-gt_::α-gt* after propagation in *E. coli* MG1655 (RM-positive) strain. Among the four phages, the percentage of 5-hmC that were α-glucosylated or β-glucosylated remained the same to that observed earlier (Figs 4E and 5F). These results further support the conclusion that the decreased virulence of phage T4∇P*_β-gt_::α-gt* was indeed due to a decrease in the percentage of overall glucosylation.

Taken together, these results demonstrate that T4 α-GT and β-GT have evolved to be able to individually glucosylate at least 80% of all 5-hmC bases in the T4 genome, which is sufficient for complete protection from 5-hmC targeting type IV RM systems and is therefore not the explanation for the presence of two GTs. The reduced virulence in RM-positive strain compared to RM− strain, of phage T4∇P*_β-gt_::α-gt* but not of phage T4∇P*_α-gt_:: β-gt*, reinforces the conclusion that the low β-glucosylation in wild-type T4 is due to the greater strength of P*_α-gt_* compared to P*_β-gt_*.

### β-GT is essential for simultaneous evasion of RM and Brig1 defense systems

Despite the redundancy ensuring complete protection against type IV RM systems in the event of loss of a GT, we reasoned that encoding two GTs could be beneficial against other defense systems targeting glycosylated DNA. A DNA glycosylase-based host defense system, Brig1, was recently shown to provide immunity against T-even phages [26] by generating abasic sites in DNA containing 5-gmC with α-glycosidic linkages. The authors showed that phage T4 was able to produce escaper mutants with mutations in *α-gt* in the presence of cosmid-borne Brig1 in the *E. coli* EC100 strain devoid of the type IV RM systems targeting 5-hmC (Supplementary Fig. S14).

**Figure 6.**
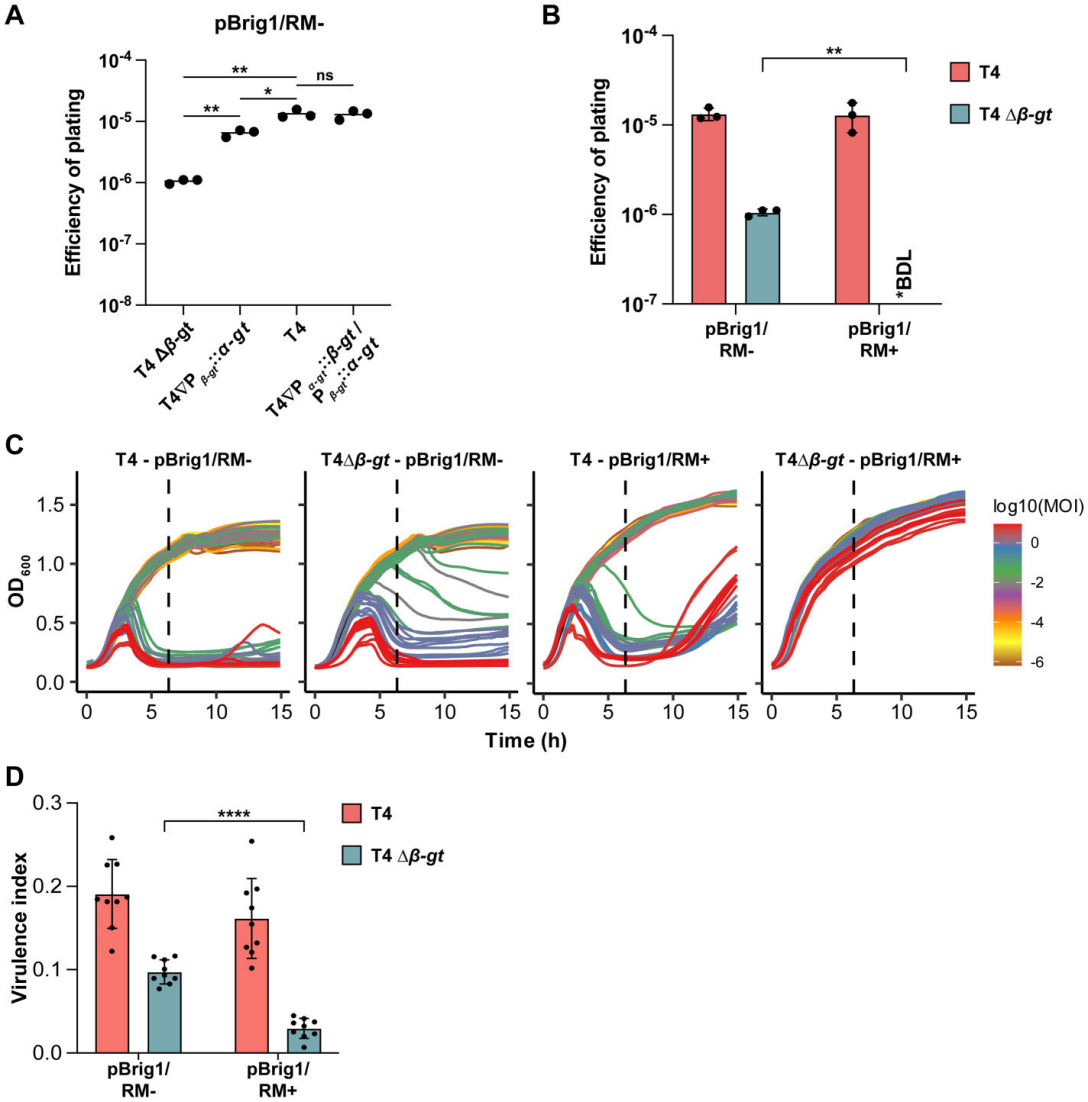
Role of β-GT in protection against Brig1. (**A**) The frequency of Brig1 escapers estimated as the EOP of phages T4Δ*β-gt*, T4∇P*_α-gt_::β-gt*, T4, and T4∇P*_α-gt_::β-gt*/P*_β-gt_::α-gt* on *E. coli* DH10B carrying a *brig1* expressing plasmid. The horizontal line represents the mean value of three biological replicates. A two-tailed unpaired *t*-test of the EOP values was used to calculate the *P-*values; **P < .01, *P < .05, ns: not significant. (**B**) Frequency of Brig1 escapers (EOP) of phages T4 and T4Δ*β-gt* in *E. coli* strains DH10B (RM−) and MG1655 (RM+), both carrying a *brig1* expressing plasmid. *BDL = below detection limit. Bars represent the mean of three biological replicates. A two-tailed unpaired *t*-test of the EOP values was used to calculate the *P-*values; **P < .01. (**C**) Growth curves of *E. coli* EC100 (RM−) and *E. coli* MG1655 (RM+) infected with phages T4 or T4Δ*β-gt* over a range of MOIs between 10^2^ and 10^−6^. Uninfected culture is represented in black. (**D**) Virulence index of the respective phages, as calculated from the growth curves shown in panel (C), up to 7 h post-infection. Bars represent the mean of nine biological replicates and error bars represent standard deviation. A two-tailed unpaired *t*-test of the virulence index values was used to calculate the *P-*values; ****P < .0001.

To understand the influence of varying levels of α-glucosylation on the anti-phage activity of Brig1, we determined the escaper frequency (*α-gt* inactivating mutants) on a RM-negative strain encoding Brig1 across a range of phage mutants with varying levels of α-glucosylation. Phages T4Δ*β-gt* and T4∇P*_β-gt_::α-gt*, both lacking β-GT and exhibiting higher α-glucosylation levels than wild-type T4, showed lower escaper frequencies than the WT, with T4Δ*β-gt* showing the lowest frequency and the higher α-glucosylation levels. The presence of β-GT, which consequently lowers α-glucosylation levels, resulted in a further two-fold higher frequency of escaper mutants in the wild-type T4 and T4∇P*_α-gt_::β-gt*∇P*_β-gt_::α-gt* phages (Fig. 6A). Hence, decrease in the α-glucosylation levels increases the escaper frequency until saturation is reached at low α-glucosylation levels. These findings demonstrate that in the absence of type IV RM systems, the competition between β-GT and α-GT increases the ability of T4 to escape Brig1 by lowering the α-glucosylation levels.

The formation of abasic sites during Brig1 activity results in escapers that are likely to contain single base pair deletions/insertions or substitution mutations, rather than large deletions typical of escapers generated through mini-homology-mediated recombination [59]. To confirm this, we analyzed the *α-gt* locus from escapers of Brig1 in comparison to escapers from CRISPR–Cas-targeting of the same locus. Indeed, none of the 29 escapers from Brig1 targeting showed any difference in size, relative to the coding region from the wild-type phage, as opposed to the escapers of CRISPR–Cas targeting (Supplementary Fig. S15 and Supplementary Table S4).

Next, we investigated the role of T4 β-GT in a situation where the host encodes both DNA glycosylase like Brig1 and RM systems. In wild-type T4, Brig1 escaper frequency was independent of the presence of RM systems (Fig. 6B). On the other hand, phage T4Δ*β-gt* was unable to generate escaper mutants in the presence of both RM and Brig1 (Fig. 6B). To quantify the consequences of these results on a population level, we estimated the virulence index of phage T4 and T4Δ*β-gt* in hosts encoding Brig1 either in the absence or presence of RM systems. T4 virulence was independent of the presence of RM systems but phage T4Δ*β-gt* was completely unable to propagate in the presence of both RM and Brig1 (Fig. 6C and D, and Supplementary Fig. S16). Based on these results we can conclude that in the presence of type IV RM systems, β-GT enables the survival of α-GT-inactivated Brig1 escapers by adequately glucosylating phage DNA.

Together, these results show that the antiphage activity of Brig1 and ensuing phage killing is partly dependent on the amount of α-glucosylation within the phage DNA. We also show that the survival of escaper T4 mutants in a host strain encoding type I and type IV RM systems along with Brig1 is entirely dependent on the phage encoding an alternative modification system such as β-GT. These results also demonstrate that in the presence of the target GT alone, DNA glycosylase combined with RM systems can completely prevent emergence of phage escaper mutants.

## Discussion

DNA hypermodification in phages is a general counter defense strategy against host defense systems whose mode of action is dependent on DNA sequence recognition, such as CRISPR–Cas and RM systems. Although it was known that bacteriophage T4 encodes two GTs with similar functions, the mechanisms underlying their respective activities and the advantage of this functional redundancy had never been thoroughly investigated.

In phage T4, the cytosines are pre-replicatively hydroxymethylated and subsequently glucosylated to the fullest extent. We show that α-GT and β-GT contribute 66% and 33%, respectively, to total glucosylation, consistent with the prior estimates of 60% and 40%, respectively [17, 19]. Competition assay involving the two GTs demonstrated that, under *in vitro* conditions, β-GT exhibited greater enzymatic activity than α-GT, which contrasts with its lower contribution to glucosylation observed *in vivo* in wild-type T4. It has been previously hypothesized that the higher *in vivo* contribution of α-GT was mainly due to its interaction with the T4 replication machinery mediated by the sliding clamp protein. Indeed, under *in vitro* conditions, we have shown that β-GT did not interact with the T4 sliding clamp protein, T4 gp45, and confirmed that α-GT co-purified with T4 gp45. Rather than the differences in binding affinity of the GTs with T4 gp45, thanks to experiments of promoter switching in T4 genome and fluorescence microscopy, we show that the proportion of cytosines undergoing α- or β-glucosylation is significantly modulated by the transcriptional and translational activities associated with each GT, together with their enzymatic activity.

Despite strict size constraints on phage genomes, why do T4 and other closely related phages encode both α-GT and β-GT? We show that deleting either *gt* did not affect phage fitness, even in the presence of type IV RM systems targeting 5-hmC-containing DNA. Moreover, the observation that, in the presence of α-GT alone, 80% of 5-hmC glucosylation fully protect phages against *E. coli* type IV systems indicates that this degree of glucosylation is sufficient for full protection. Interestingly, we observed that the mutant phage T4∇P*_β-gt_::α-gt*, with α-GT expressed from the *β-gt* promoter, makes smaller plaques and has a lower virulence index than other phages. The percentage of glucosylated 5-hmC was estimated to be 75%, a proportion only slightly lower to that observed in the single *β-gt* deletion genotype. This suggest that 80% of glucosylation is the absolute minimal threshold necessary to protect against the *E. coli* McrBC/McrA RM systems.

Furthermore, Lunt and colleagues have observed restricted glucosylation by α-GT in sequences with consecutive hmC flanked by purine nucleotides [60, 61]. Hence, it is likely that the 80% glucosylation observed upon expression of α-GT alone from its native promoter reflects an enzymatic restriction rather than limited expression from P*_α-gt_*. In conclusion, in the wild-type phage, competition between the two enzymes ensures balanced distribution of the two types of linkages, but when expressed alone each enzyme ensures adequate glucosylation and complete protection of phage DNA.

Here, we also demonstrate that growth of phages encoding a single GT is entirely abolished in hosts encoding defense systems, such as Brig1, that target specific stereoisomers of 5-gmC, in addition to classical RM systems. On the contrary, phages encoding two different GTs can generate escape mutants at high frequency (1 × 10^−5^). Indeed, in the presence of a single GT, mutations inactivating α-GT leads to phage DNA targeting by type IV RM systems and are therefore lethal. By contrast, in the presence of two GTs with different stereospecificity, mutations in *α-gt* are offset by β-glucosylation, ensuring survival of escape mutants with inactivating mutations in *α-gt*. Furthermore, among hosts encoding only the DNA glycosylase defense system, the presence of non-targeted GT(s) promotes a higher rate of escaper generation. Our results emphasize the benefits of GT diversity in phage genomes, and in the diversity of the types of modification on a more general level. These findings also underscore a notable drawback inherent to DNA glycosylase-based defense systems, such as Brig1, in that their activity invariably results in escaper phages in the absence of co-occurring defense systems. Analysis of Brig1 escapers across the *α-gt* locus showed that the mutants comprised only point mutations (single base pair insertions or deletions and substitutions), with none of the mutants showing larger deletions across or within *α-gt*, in contrast to what is observed in escapers from CRISPR–Cas systems [59]. This constitutes an advantage for Brig1 escapers, as point mutations are reversible, offering the possibility to recover functional genes. Hence, encoding two GTs whose products are stereoisomers confers an evolutionary advantage when faced with a stereoisomer-specific host defense system.

The frequent association of TAGT homologs with both α-GT and β-GT indicates that either the former does not differentiate between its substrate’s linkage type or that there are two distinct kinds of TAGT among its homologs. Furthermore, the presence in 288 out of 753 phage genomes of solitary TAGT homologs, in the absence of both α-GT and β-GT, indicates that these phages potentially encode novel transferases, which forms the basis for additional glucosyl modifications. The not-so-limited prevalence of β-GT and arabinosylation clusters among phages [30], along with the identification of diverse DNA hypermodification enzymes from metagenomic DNA [62], suggests that Brig1 is likely not the only DNA glycosylase-based host anti-phage defense system. Similarly, it is also likely that the yet-to-be defined function of the TAGTs lies in their ability to overcome DNA glycosylases that indiscriminately target products of both α-GT and β-GT. To an extent, this has been demonstrated in Bas46 phage, where deletion of the arabinosyltransferase Aat resulted in susceptibility to a plasmid-encoded defense system [30]. Overall, only 6 out of the 753 phage genomes, including phage T4 and its close relatives, encoded both α-GT and β-GT. The limited co-occurrence of α-GT and β-GT might indicate that the adaptation of DNA glycosylase defense system by the hosts and the counter measure of encoding multiple hypermodification enzymes by phages is a relatively recent innovation.

Encoding multiple hypermodification enzymes such as GTs along with dCTPase, thymidylate synthase, IPI, IPII, and IPIII is a great example of continuous antagonistic evolution of defense and counter defense systems. Phages hydroxymethylate their cytosines in response to sequence-specific targeting by the host type I RM system [22], which, in turn, makes them susceptible to the type IV RM system [5, 23]. This vulnerability is then countered by sugar modification, such as glucosylation or arabinosylation, of the cytosines [17, 18, 30]. These modifications not only confer resistance to type IV RM systems but also enable the phage to evade targeting by certain types of CRISPR–Cas systems [5–10, 30]. Other type IV RM systems such as GmrSD are capable of indiscriminately targeting the DNA modified by the primary GTs, eliciting the evolution of phage-encoded cognate inhibitors, IPI, IPII, and IPIII [24, 25, 27]. Subsequently, when hosts evolve stereoisomer-specific DNA glycosylases such as Brig1 [26, 31], instead of evolving a cognate inhibitor, phage T4 has acquired a variant GT enzyme with different stereospecificity, β-GT, to diversify the distribution of the glycosidic linkages in its genome.

## Supplementary Material

gkag531_Supplemental_Files

## Data Availability

The data underlying this article are available in the article and in its online supplementary material.
