# Supplementary material for: A pair of DNA glucosyltransferases elevate counter-defense in bacteriophage T4
Source: Nucleic Acids Res. 2026 May 28;54(10):gkag531. doi: 10.1093/nar/gkag531 (PMC13216743; doi:10.1093/nar/gkag531)
Supplement: gkag531_Supplemental_Files [file gkag531_supplemental_files.zip › Supplementary information.pdf]

Supplementary information for

A Pair of DNA Glucosyltransferases Elevate Counter-defense in Bacteriophage T4

Luis Ramirez-Chamorro[1], Frédéric Bonhomme[2], Anton Lukas Ipsen Wolff[3], Mathieu Stouf[1], François Lecointe[1], Marcel Hollenstein[4], Mart Krupovic[5], Marianne De Paepe[1] & Yuvaraj Bhoobalan-Chitty[1,3,*]

Affiliations:

[1] Université Paris-Saclay, INRAE, AgroParisTech, Micalis Institute, 78350 Jouy-en-Josas, France

[2] Institut Pasteur, Université Paris Cité, CNRS UMR3523, Department of Structural Biology and Chemistry, Unité de Chimie Biologique Epigénétique, 28, rue du Docteur Roux, 75724 Paris Cedex 15, France

[3] Department of Biology, University of Copenhagen, Copenhagen N, Denmark

[4] Institut Pasteur, Université Paris Cité, CNRS UMR3523, Department of Structural Biology and Chemistry, Laboratory for Bioorganic Chemistry of Nucleic Acids, 28, rue du Docteur Roux, 75724 Paris Cedex 15, France

[5] Institut Pasteur, Université Paris Cité, Cell Biology and Virology of Archaea Unit, Paris, France

*Address correspondence to Yuvaraj Bhoobalan-Chitty, yuvarajb@bio.ku.dk

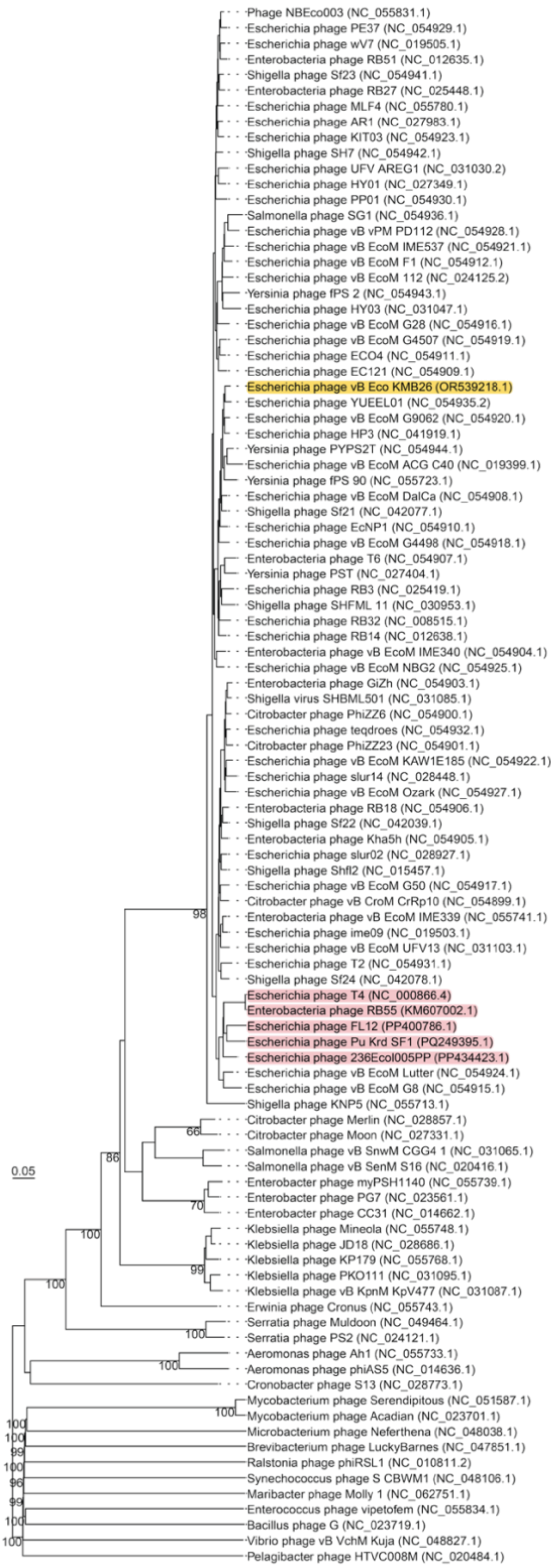

Supplementary Figure 1: Phylogenetic tree based on the whole-genome comparison of phages encoding GTs. The phages highlighted in red encode both primary GTs and the phage highlighted in yellow encodes all three GTs. The Genome-BLAST Distance Phylogeny pseudo-bootstrap support values from 100 replicates are shown above the branches. The tree was generated using the VICTOR web service (https://ggdc.dsmz.de/victor.php) (1), with branch lengths scaled according to the nucleotide distance formula (D0).

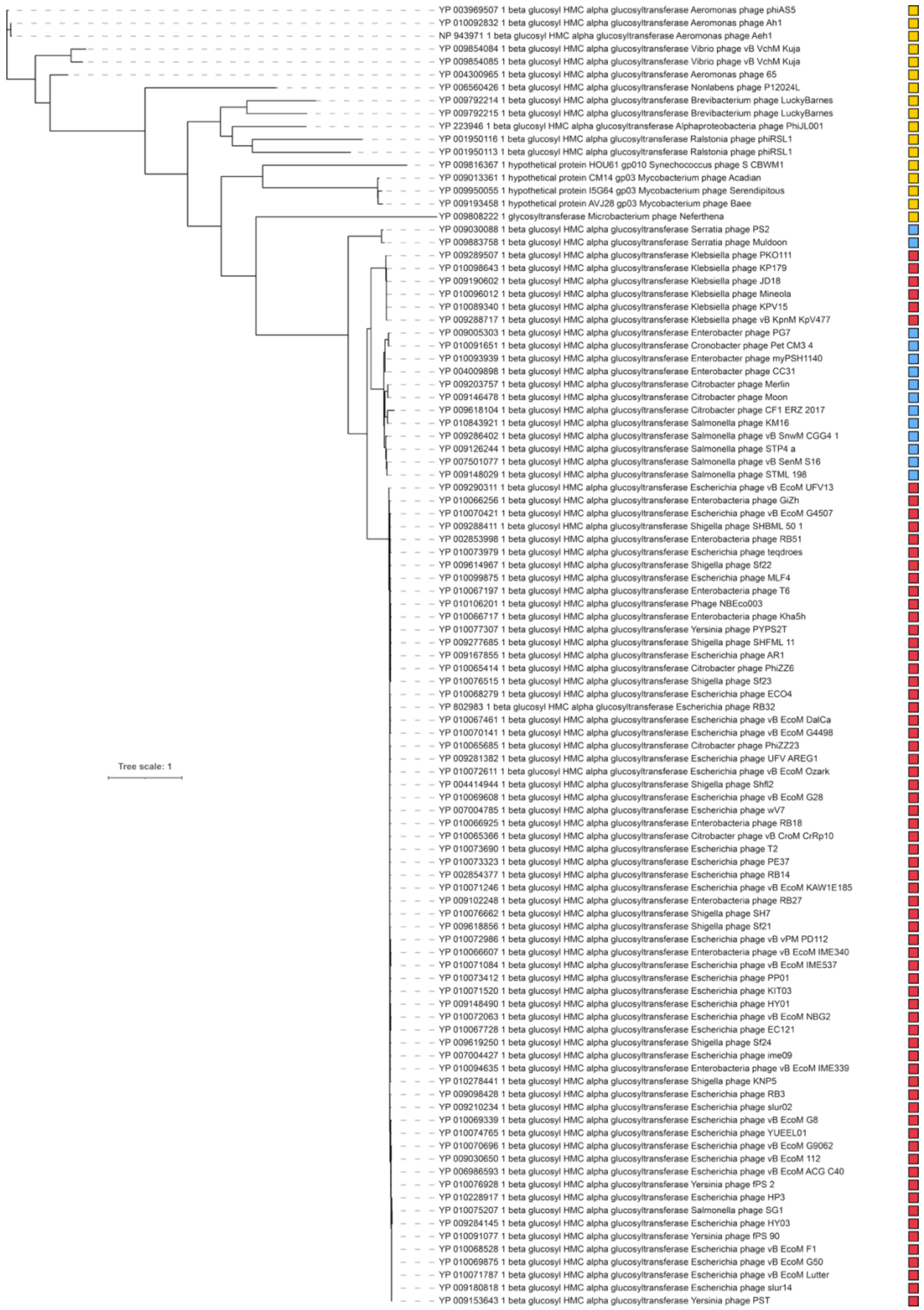

Supplementary Figure 2: Maximum likelihood phylogeny of the secondary glucosyltransferase (β-glucosyl-HMC-α/β-GT). The box adjacent to each leaf label indicates co-occurrence with either α-GT (red) or β-GT (blue), or lack of association with a primary GT (yellow). The tree was generated using the NGphylogeny.fr (2) web service.

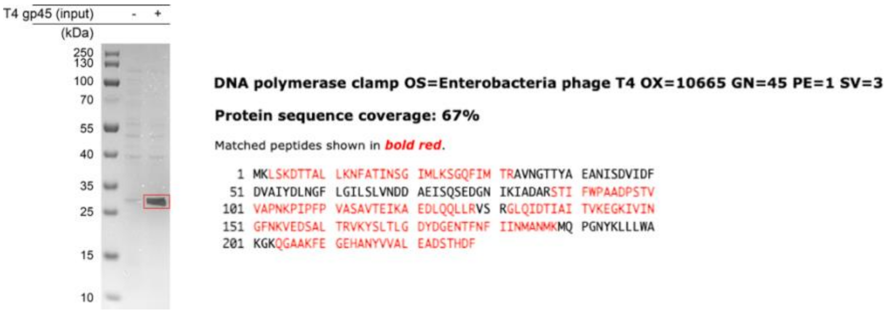

Supplementary Figure 3: Validation of un-tagged T4 gp45 expression in *E. coli* BL21(DE3). Lane 1 and lane 2 correspond to the total cell extract of *E. coli* BL21(DE3) with un-tagged T4 gp45, before and after induction with IPTG. The induced protein (intense band) corresponding to the size of the un-tagged sliding clamp (T4 gp45, 24858.32 Da) monomer, was excised from the gel (shown inside the red box) and the protein's identity confirmed by LC-MS/MS (right panel).

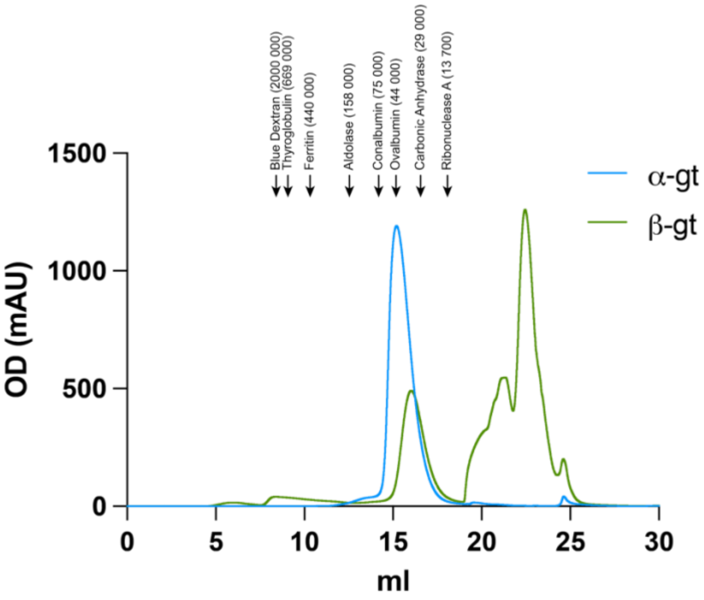

Supplementary Figure 4: Size-Exclusion Chromatography of C-terminally histidine tagged α-GT and β-GT. Elution profiles of histidine tagged α-GT (blue) and β-GT (green) obtained upon separation of the proteins on a Superdex 200 Increase 10/300 GL column. The absorbance was monitored at 280 nm. The peak elution position of each standard protein is indicated as an arrow, along with their theoretical molecular weights (Da) in brackets. The molecular weights of α-GT and β-GT, based on their peak elution volumes, were estimated to be 48.36 kDa and 33.61 kDa, respectively.

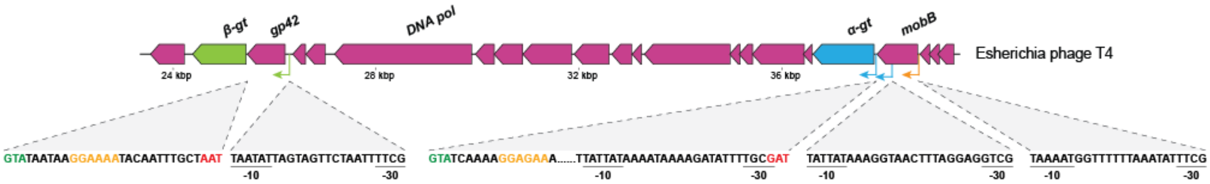

Supplementary Figure 5: Illustration of regulatory elements associated with *α-gt* and *β-gt*. The -30 and -10 regions of the middle promoter motifs (underlined) and the Shine-Dalgarno site (highlighted in yellow) associated with *α-gt* and *β-gt* or their co-expressed upstream gene is depicted. The start and stop codon of overlapping gene are highlighted in green and red, respectively.

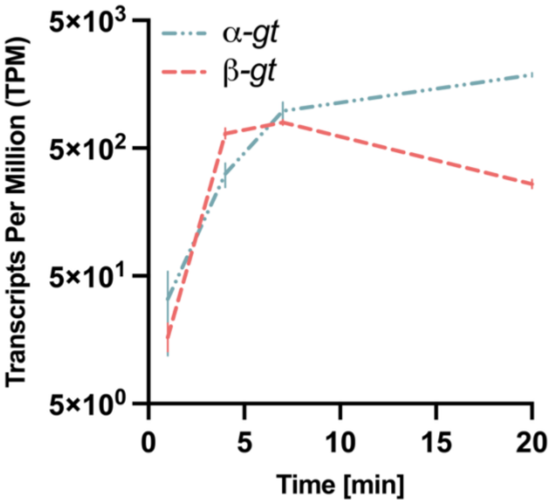

Supplementary Figure 6: Transcript levels of *α-gt* and *β-gt* during the T4 infection cycle. Average of the three replicates, each normalized with the total reads corresponding to phage transcripts, was plotted against time post infection. Raw data was obtained from Wolfram-Schauerte and Pozhydaieva et al., 2022 (3).

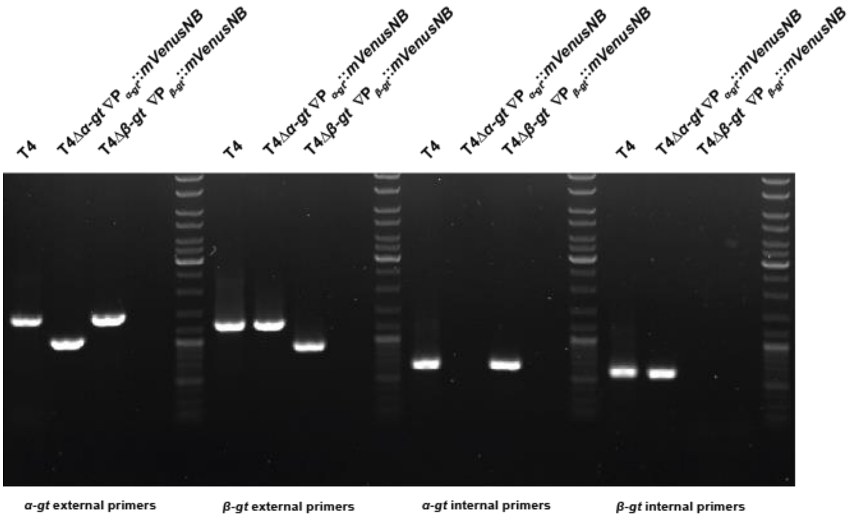

Supplementary Figure 7: PCR confirmation of *mVenusNB* insertion in phages T4Δ*α-gt*∇P$_{α\text{-}gt}$::*mVenusNB* and T4Δ*β-gt*∇P$_{β\text{-}gt}$::*mVenusNB*. The insertion of *mVenusNB* in the mutant phages T4Δ*α-gt*∇P$_{α\text{-}gt}$::*mVenusNB* and T4Δ*β-gt*∇P$_{β\text{-}gt}$::*mVenusNB* is confirmed with internal primers and external primers (listed in Supplementary Table S8) for for *α-gt* and *β-gt*.

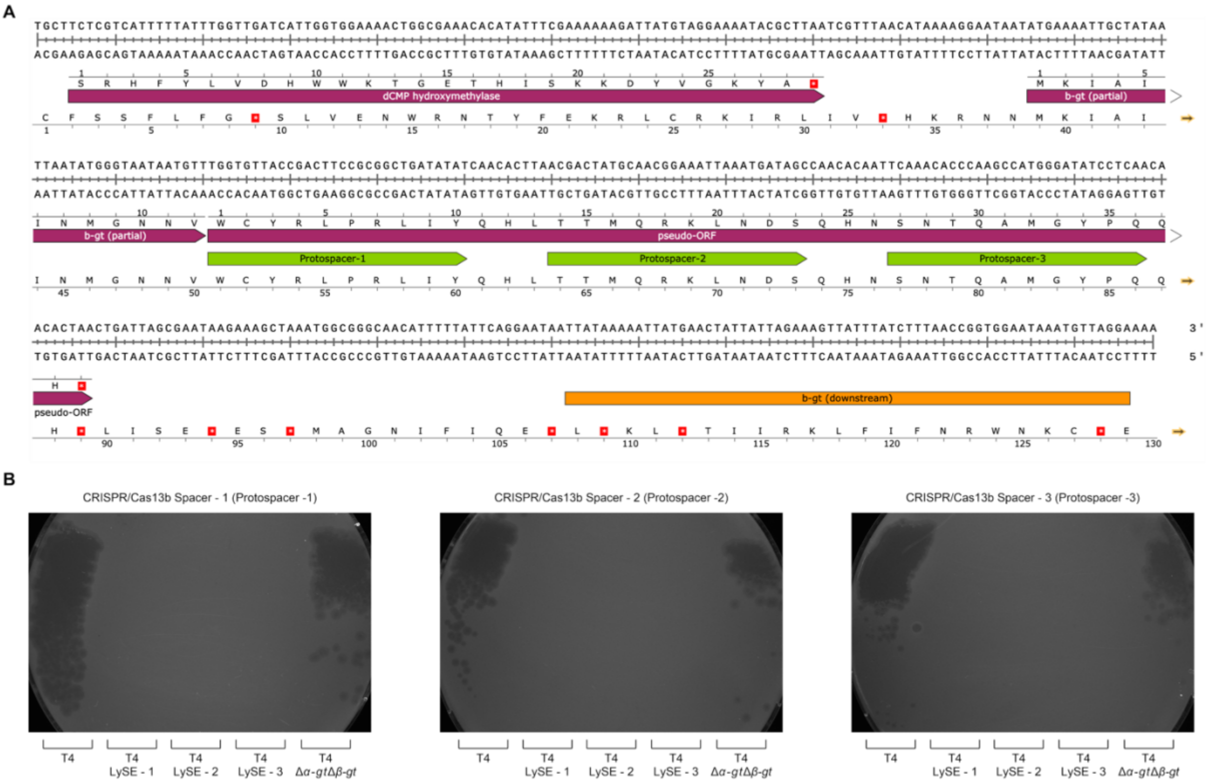

Supplementary Figure 8: Genome editing strategy for swapping genomic locations of *α-gt* and *β-gt*. A The coding region including the pseudo-ORF, in-frame, with the partial *β-gt* sequence is shown. The protospacers for Cas13b targeting are shown as arrows (in green). The upstream (*gp42* and partial *β-gt*) and downstream (*β-gt* downstream) sequences, necessary for recombination into the wild-type T4, are shown in purple and orange respectively. **B** Susceptibility of the phage T4 LySE (3 clonal copies) to CRISPR-Cas13b in *E. coli* DH10B (RM-) is shown. Left panel: Cas13b spacer complimentary to protospacer–1, middle panel: spacer complimentary to protospacer–2 and right panel: spacer complimentary to protospacer–3. T4 wild-type and T4Δ*α-gt*Δ*β-gt*, both lacking the pseudo-ORF, are shown as controls.

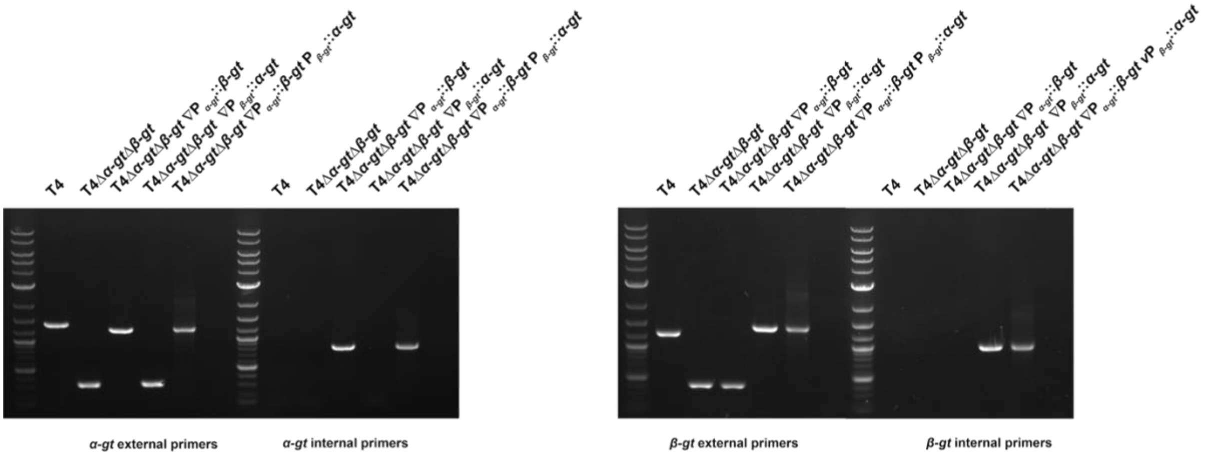

Supplementary Figure 9: PCR confirmation of the *α-gt* and *β-gt* genomic location swapping in different phage mutants. The positional swapping of *α-gt* and *β-gt*, along with their presence and absence, in the phage mutants T4 $\nabla P_{\alpha\text{-}gt}$::*β-gt*, T4 $\nabla P_{\beta\text{-}gt}$::*α-gt* and T4 $\nabla P_{\alpha\text{-}gt}$::*β-gt* $P_{\beta\text{-}gt}$::*α-gt* was confirmed with internal primers and external primers for *α-gt* and *β-gt* (listed in Supplementary Data 4). Phage T4 was used as PCR control.

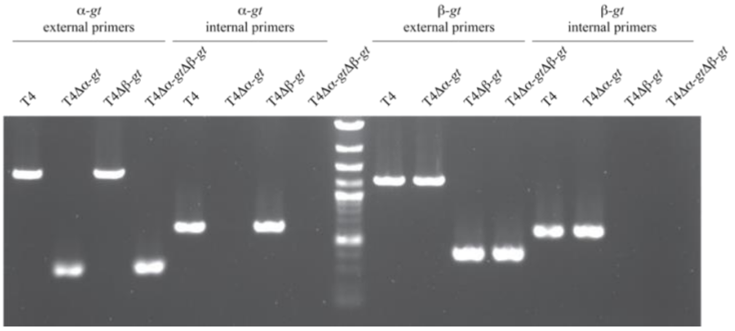

Supplementary Figure 10: PCR confirmation of the desired deletion in T4Δ*α-gt*Δ*β-gt*. The deletion of *α-gt* and *β-gt* in the mutant phage T4Δ*α-gt* Δ*β-gt* is confirmed with internal primers and external primers (listed in Supplementary Table S8) for *α-gt* and *β-gt*. Mutant phages T4Δ*α-gt* and T4Δ*β-gt*, constructed earlier (4), along with the phage T4 were used as PCR controls.

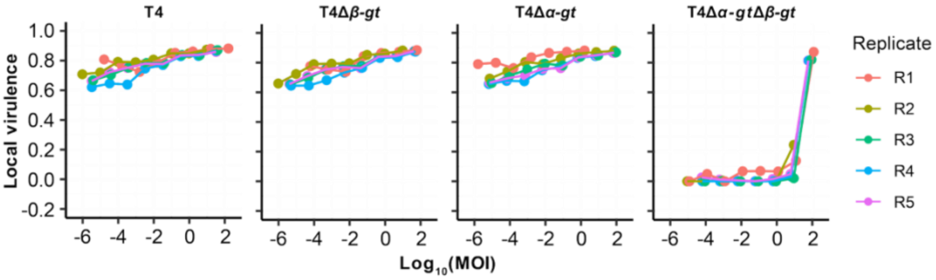

Supplementary Figure 11: Local virulence corresponding to phages T4, T4Δ*α-gt*, T4Δ*β-gt* and T4Δ*α-gt*Δ*β-gt* upon infection in *E. coli* MG1655. The local virulence for each MOI was calculated from the corresponding growth curve in Fig. 5A.

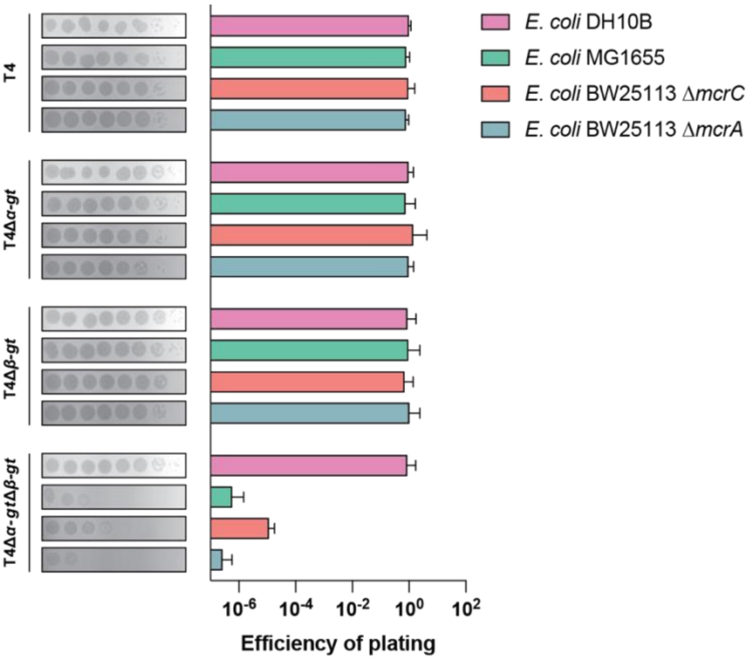

Supplementary Figure 12: Efficiency of plating of T4 wild-type, T4 Δ*α-gt*, *T4* Δ*β-gt* and T4 Δ*α-gt*Δ*β-gt* in *E. coli* DH10B, *E. coli* MG1655, *E. coli* BW25113 Δ*mcrC* and *E. coli* BW25113 Δ*mcrA*. Spot assay of serially diluted phage preps on a lawn of individual host strains. Efficiency of plating (EOP) of phage infection, as estimated from the spot assay, are from three biological replicates, represented as mean ± SD.

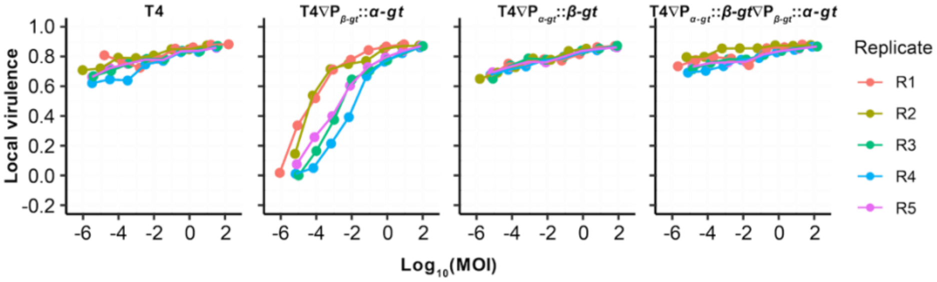

Supplementary Figure 13: Local virulence of phages T4 $\nabla P_{\alpha\text{-}gt}$::*β-gt*, T4 $\nabla P_{\beta\text{-}gt}$::*α-gt* and T4 $\nabla P_{\alpha\text{-}gt}$::*β-gt*/$P_{\beta\text{-}gt}$::*α-gt* in comparison to wild-type T4, upon infection in *E. coli* MG1655. The local virulence for each MOI was calculated from the corresponding growth curve in Fig. 5C.

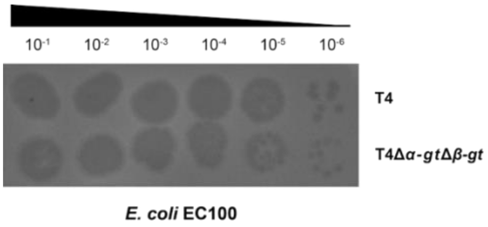

Supplementary Figure 14: *E. coli* EC100 strain lacks type IV RM systems that target 5-hmC. Spot assay of serially diluted wild-type T4 and T4 Δ*α-gt*Δ*β-gt* on a lawn of *E. coli* EC100.

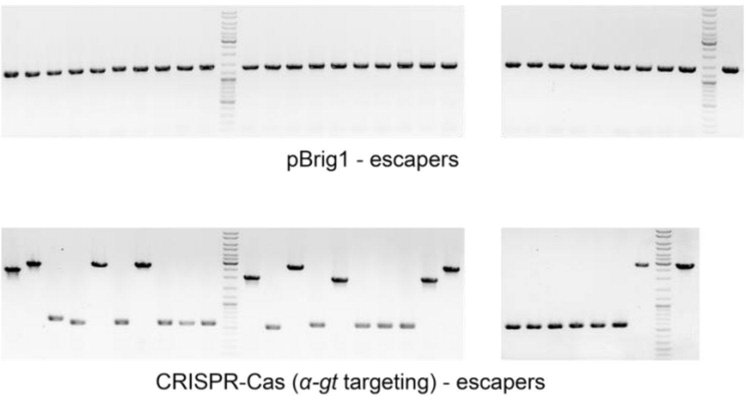

Supplementary Figure 15: PCR analysis of the *α-gt* locus in phage T4 escapers generated post targeting by Brig1 (upper panel) or the CRISPR-Cas13b system (with a spacer complementary to the transcript of *α-gt*).

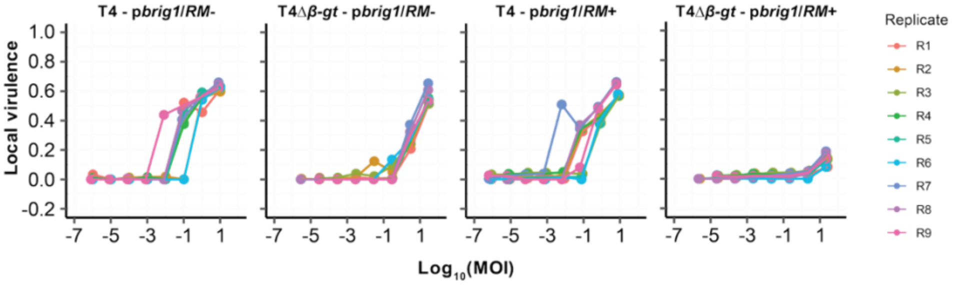

Supplementary Figure 16: Local virulence corresponding to phage T4 and T4Δ*β-gt* upon infection in *E. coli* EC100 (RM-) and *E. coli* MG1655 (RM+). The local virulence for each MOI was calculated from the corresponding growth curve in Fig. 6C.

References:

1. Meier-Kolthoff, J.P. and Goker, M. (2017) VICTOR: genome-based phylogeny and classification of prokaryotic viruses. *Bioinformatics*, **33**, 3396-3404.
2. Lemoine, F., Correia, D., Lefort, V., Doppelt-Azeroual, O., Mareuil, F., Cohen-Boulakia, S. and Gascuel, O. (2019) NGPhylogeny.fr: new generation phylogenetic services for non-specialists. *Nucleic Acids Res*, **47**, W260-W265.
3. Wolfram-Schauerte, M., Pozhydaieva, N., Viering, M., Glatter, T. and Hofer, K. (2022) Integrated Omics Reveal Time-Resolved Insights into T4 Phage Infection of E. coli on Proteome and Transcriptome Levels. *Viruses*, **14**.
4. Bhoobalan-Chitty, Y., Stouf, M. and De Paepe, M. (2024) Genetic manipulation of bacteriophage T4 utilizing the CRISPR-Cas13b system. *Front Genome Ed*, **6**, 1495968.
